# Complex Population Structure and Virulence Differences among Serotype 2 *Streptococcus suis* Strains Belonging to Sequence Type 28

**DOI:** 10.1371/journal.pone.0137760

**Published:** 2015-09-16

**Authors:** Taryn B. T. Athey, Jean-Philippe Auger, Sarah Teatero, Audrey Dumesnil, Daisuke Takamatsu, Jessica Wasserscheid, Ken Dewar, Marcelo Gottschalk, Nahuel Fittipaldi

**Affiliations:** 1 Public Health Ontario, Toronto, Ontario, Canada; 2 Groupe de Recherche sur les Maladies Infectieuses du Porc, Faculté de Médecine Vétérinaire, Université de Montréal, St-Hyacinthe, Quebec, Canada; 3 Bacterial and Parasitic Diseases Research Division, National Institute of Animal Health, National Agriculture and Food Research Organization, Tsukuba, Japan; 4 The United Graduate School of Veterinary Sciences, Gifu University, Gifu, Japan; 5 Department of Human Genetics, McGill University and Génome Québec Innovation Centre, Montreal, Quebec, Canada; 6 Department of Laboratory Medicine and Pathobiology, Faculty of Medicine, University of Toronto, Toronto, Ontario, Canada; Centers for Disease Control & Prevention, UNITED STATES

## Abstract

*Streptococcus suis* is a major swine pathogen and a zoonotic agent. Serotype 2 strains are the most frequently associated with disease. However, not all serotype 2 lineages are considered virulent. Indeed, sequence type (ST) 28 serotype 2 *S*. *suis* strains have been described as a homogeneous group of low virulence. However, ST28 strains are often isolated from diseased swine in some countries, and at least four human ST28 cases have been reported. Here, we used whole-genome sequencing and animal infection models to test the hypothesis that the ST28 lineage comprises strains of different genetic backgrounds and different virulence. We used 50 *S*. *suis* ST28 strains isolated in Canada, the United States and Japan from diseased pigs, and one ST28 strain from a human case isolated in Thailand. We report a complex population structure among the 51 ST28 strains. Diversity resulted from variable gene content, recombination events and numerous genome-wide polymorphisms not attributable to recombination. Phylogenetic analysis using core genome single-nucleotide polymorphisms revealed four discrete clades with strong geographic structure, and a fifth clade formed by US, Thai and Japanese strains. When tested in experimental animal models, strains from this latter clade were significantly more virulent than a Canadian ST28 reference strain, and a closely related Canadian strain. Our results highlight the limitations of MLST for both phylogenetic analysis and virulence prediction and raise concerns about the possible emergence of ST28 strains in human clinical cases.

## Introduction

*Streptococcus suis* is a major swine pathogen responsible for septicemia, meningitis and other diseases in swine that often result in severe economic losses to the porcine industry [1]. *S*. *suis* is also an emerging zoonotic agent [2]. Two outbreaks of human *S*. *suis* disease occurred in China in 1998 and 2005, affecting hundreds of people and killing more than forty [3]. Relatively recent reports found that this pathogen is the first and second-most commonly reported cause of adult streptococcal meningitis in Vietnam and Thailand, respectively [4, 5]. On the other hand, in European countries, human *S*. *suis* disease has never been associated with large outbreaks, and has mostly affected workers in the swine industry [6]. Relatively very few cases of human *S*. *suis* disease have been reported in North America [6]. Most cases of animal and human *S*. *suis* infection are caused by serotype 2 strains [7]. Interestingly, the percentage of *S*. *suis* serotype 2 strains recovered from diseased pigs has historically been lower in North America than in other parts of the world [8].

Increased research in recent years has identified a myriad of virulence factors involved in the pathogenesis of infection of *S*. *suis* serotype 2 [9]. *S*. *suis* strains belonging to serotype 2 can be divided by multilocus sequence typing (MLST), into at least 16 sequence types (STs) with closely related STs grouped into ST clonal complexes (CCs) [7, 10]. Most virulence studies have been carried out with a limited number of ST1 and ST7 serotype 2 strains, which were predominately isolated from diseased pigs in the Netherlands, France, Spain, the United Kingdom, and China [7, 9]. While ST1 and ST7 strains are more prevalent in these and a few other countries, previous work has shown that in North America ST25 and ST28 strains predominate, accounting for 44% (ST25) and 51% (ST28) of all strains investigated [11]. The *S*. *suis* MLST scheme is based on the sequence of seven housekeeping genes [10]. Thus, a significant amount of information such as DNA polymorphisms occurring in other parts of the genome, and gene content variation encoded in mobile genetic elements, is not captured by this typing method. Virulence studies showed that one ST28 strain from Canada was significantly less virulent than ST1 and ST25 strains [11]. However, little is known about variation within the ST28 group. Assessing this intra-ST variation is important. For example, earlier work showed that ST28 *S*. *suis* strain 1330 was avirulent in both mice and swine [12]. More recently, it was shown that an ST28 strain isolated from the tonsils of an asymptomatic pig in China had very low virulence in a swine model of infection [13]. However, ST28 strains are often isolated from diseased swine in China and Japan [14–16], and at least four human ST28 cases have been reported in Thailand and Japan [17–19]. Moreover, some have speculated that while porcine ST28 *S*. *suis* infections in North America are most often associated with a concomitant viral infection, in some Asian countries ST28 strains may be the primary pathogen [1]. These findings support the hypothesis that not all ST28 strains have the same virulence potential. They also raise doubts about the universal value of previous virulence studies conducted with only one ST28 organism.

Here, we sought to use genomics to analyze the population structure of a collection of 51 *S*. *suis* serotype 2 ST28 strains isolated in four different countries (Canada, the United States of America, Japan, and Thailand), and to investigate virulence traits of selected ST28 strains. We report a complex population structure among ST28 strains, which were shown to belong to at least 5 different clades following whole-genome-single nucleotide polymorphism (SNP) analysis. We also show important virulence differences between some of these genetic groups.

## Materials and Methods

### Strains, culture conditions and DNA preparation

A total of 50 *S*. *suis* serotype 2 ST28 strains isolated from diseased pigs (20 from Canada, 15 from USA, and 15 from Japan) and one strain isolated from a human case in Thailand, collected from 1990 to 2011, were used (S1 Table). These strains had previously been serotyped, and typed by MLST using standard procedures [10, 20]. Strains were cultured on Columbia blood agar plates containing 5% sheep blood, and grown at 37°C with 5% CO_2_. Liquid cultures were grown in Todd-Hewitt broth supplemented with 0.2% yeast extract. DNA was prepared from 5 ml of overnight *S*. *suis* cultures using the QIAamp DNA minikit (Qiagen, Toronto, ON, Canada) following the manufacturers’ protocol for Gram positive organisms.

### Whole-genome sequencing and closure of a reference ST28 genome

Whole genome sequencing libraries were prepared for all 51 isolates using Nextera XT kits (Illumina, San Diego, CA, USA) and sequenced as paired-end reads with either a HiSeq 2500 (101 bp + 101 bp) or a MiSeq (150 bp + 150 bp) instrument. Parsing of the multiplexed sequencing reads and removal of barcode information was done using onboard software. Short-read sequences have been deposited in the Sequence Read Archive under accession number SRP058193. MLST STs were derived directly from the short-read data using SRST2 software [21] and used to confirm previous MLST results. We next sequenced to closure the genome of strain NSUIS002 using SMRT sequencing (Pacific Biosciences, Menlo Park, CA, USA). This strain had previously been named 1088563 and was selected for genome closure because 1) it belonged to the more prevalent Canadian group and 2) virulence data in a murine model of infection had previously been obtained [11]. Briefly, two SMRT cells of sequence were run, generating 51,367 reads exceeding 3 kb in length (average read length of 6.4 kb; 146X coverage for reads >3 kb). Next, we used HGAP v2 [22] to correct the long reads and Celera Assembler 7.0 [23] to assemble the corrected reads, followed by two rounds of polishing with Quiver (https://github.com/PacificBiosciences/GenomicConsensus). The coverage of the final assembly in reads >3 kb was 146X. To assess base-calling accuracy in the Pacific Biosciences assembly, Illumina short-reads were aligned to the assembly using BLAT [24]. The genome assembly was completely concordant with full length perfectly aligning Illumina short-reads. The genome was formatted to begin at the first nucleotide of the intergenic region immediately preceding gene *dnaA*, encoding a chromosomal replication initiation protein. The finalized genome was annotated using Prokka [25] and deposited in GenBank under Accession number CP011419.

### Core-genome, assessment of recombination, phylogenetic analysis, and antibiotic resistance genes

The A5 pipeline was used for *de novo* assembly of Illumina sequenced strains [26]. Obtained contigs were ordered relative to the NSUI002 reference genome using Progressive Mauve [27]. Then, pseudochromosomes were created for the remaining 50 strains by concatenating the ordered contigs using the sequence NNNNNCATTCCATTCATTAATTAATTAATGAATGAATGNNNNN, which introduces start and stop codons in all 6 reading frames, as a separator. Pseudochromosomes were annotated using Prokka. We next defined a core chromosome following the method of de Been *et al* [28]. Briefly, InParanoid [29] and QuickParanoid (http://pl.postech.ac.kr/QuickParanoid) were used to identify ortholog gene clusters between all ST28 strains. Genes encoded in mobile genetic elements and ortholog genes varying in length by more than 9 bp were not considered. Next, for each ortholog group, the sequences were aligned using Muscle v3.7 [30] and gaps were removed using trimAl v1.2 [31]. These aligned and trimmed genes were then reassembled for each strain in the order in which the gene appeared in the NSUI002 reference. Recombination occurring in the so defined core genome was assessed using BRATNextGen [32] run with 20 iterations and 100 replicates, using a *p*-value of 0.05 as the significance cutoff. For phylogenetic analysis, SNPs relative to the genome of reference strain NSUI002 were identified for each of the 50 additional ST28 strains using VAAL [33]. A matrix file containing the genotype of all strains at each polymorphic locus was then created from the VAAL polymorphism output data using a custom script. Next, all SNPs occurring in areas of the genome not found in the above-defined core genome (i.e. those occurring in mobile genetic elements, intergenic regions, and NSUI002 genes without an ortholog in all 51 ST strains) were discarded. As well, we eliminated SNPs occurring in genes that were deemed to have undergone recombination based on BRATNextGen results. Then, for each individual strain, SNPs were concatenated in order of occurrence relative to the genome of the reference strain and converted to a multiFASTA sequence. Neighbor-joining phylogenetic trees (1,000 bootstrap replications) were generated with SplitsTree4 [34]. We used SRST2 and a database listing 1913 variants of genes encoding antimicrobial resistance [21] (https://github.com/katholt/srst2) to test for presence or absence of genetic determinants of antimicrobial resistance in the genomes of the ST28 strains. Genome visualizations were created using BRIG [35] and edited using Adobe Illustrator.

### Experimental mouse infections

We used a validated C57BL/6 murine model of infection [36]. Briefly, 75 mice (aged 6–10 weeks, Jackson Laboratory) were acclimatized to standard laboratory conditions with a 12-h light/12-h dark cycle and unlimited access to water and food. On the day of the experimental infection, five groups of 15 animals each were defined. Group 1 received a 1-ml injection of the Canadian ST28 strain NSUI002 suspension (at 1 x 10^8^ CFU), delivered using the intraperitoneal route. Groups 2, 3, 4, and 5 received the same amount of strains NSUI062, NSUI010, NSUI081, and NSUI036, respectively. Mice were monitored 3 times/day for the first 72 h and then twice daily until 14 days post-infection (pi) for clinical signs and assigned clinical scores as previously described [37]. Blood was collected 24 h and 48 h pi from the tail vein (5 μl), appropriately diluted and used to evaluate bacterial load by plating onto sheep blood agar plates and enumeration [37]. All experiments involving mice were conducted in accordance with the guidelines and policies of the Canadian Council on Animal Care. Humane endpoints approved by the Animal Welfare and Ethics Committee, Université de Montréal, were used. Animals were evaluated every 8 h during the first 72 h and twice daily after, and clinical scores were assigned. Animals presenting a score of 4 or 5 (moderately sick) were evaluated every 4 h. Mice presenting a score of 6 were evaluated every 4 h and euthanized if their score remained constant after 24 h. Mice presenting a score of 7 were immediately euthanized. Animals were euthanized using inhaled CO_2_. Animal suffering was minimized by careful and timed evaluation of animals, by closely following the scoring grid, and by immediate euthanasia, if required.

### Statistical Analysis

The R software [38] was used for statistical analysis. Differences in survival curves were assessed using log-rank test. Differences in bacteremia were assessed using ANOVA on ranks and Tukey test at 24 h and 48 h post-infection (pi). A *P*-value of less than 0.05 was used as the cutoff for significance.

## Results and Discussion

### Genome closure of reference strain NSUI002 and comparison to other *S. suis* genomes

**Fig 1:**
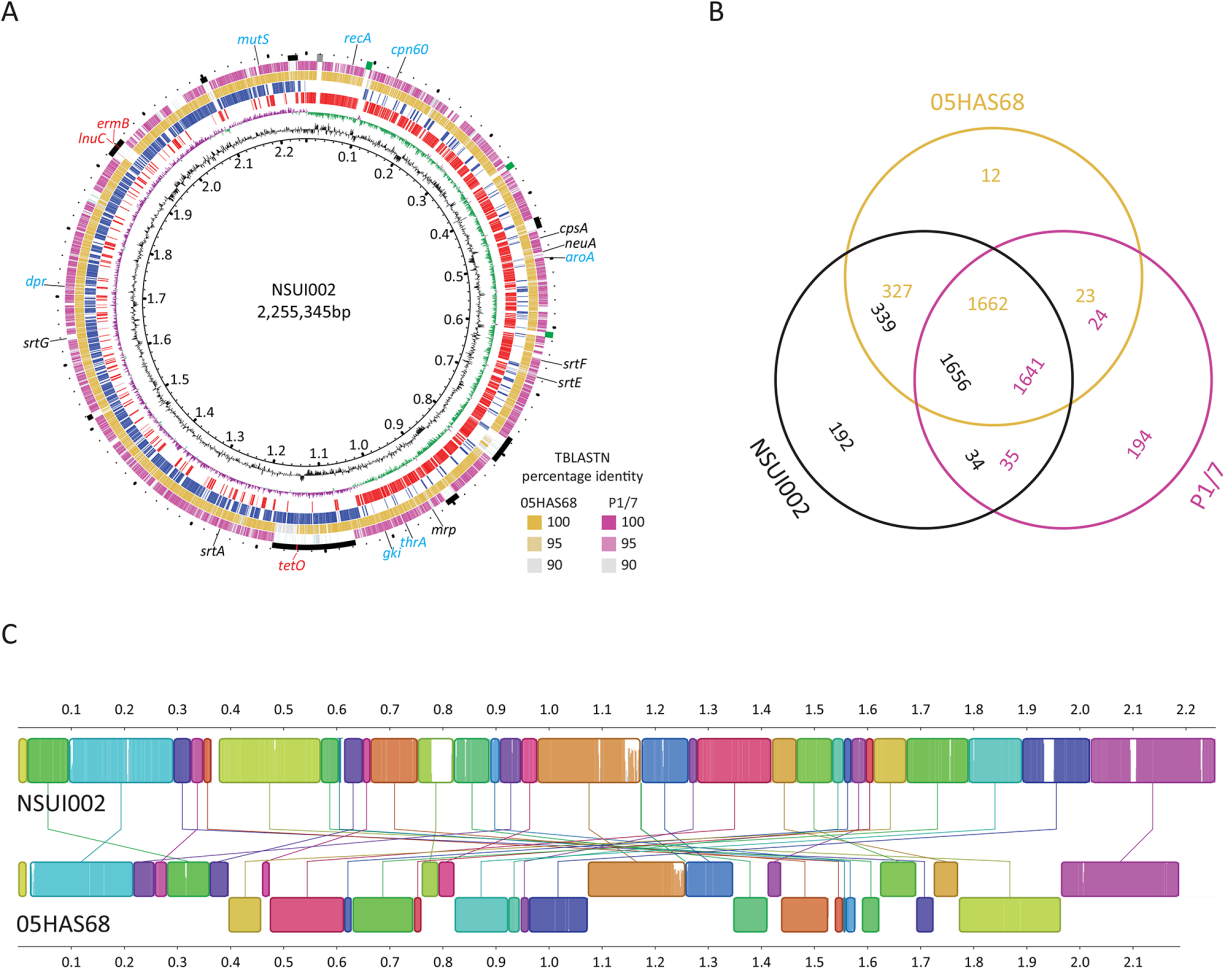
A) Genome atlas of Canadian *S*. *suis* ST28 strain NSUI002. Depicted data from innermost to outermost circles represent genome size in Mbp (circle 1); percent G+C content (circle 2); GC skew (circle 3), (G-C)/(G+C) averaged over a moving window of 10,000 bp, with excess G and excess C shown in green and purple, respectively; annotated coding sequences (CDSs) encoded on the forward/direct (circle 4, red), and reverse/complementary (circle 5, blue) chromosomal strands; TBLASTN comparisons of the CDSs predicted in ST28 strains NSUI002 and 05HAS68 (circle 6, percent identity defined in the Fig), TBLASTN comparisons of ST28 strain NSUI002 and ST1 strain P1/7 (circle 7, percent identity defined in the Fig); reference genome landmarks (circle 8): ribosomal RNAs are labeled in green; mobile genetic elements are labeled in black, genes used in the *S*. *suis* MLST scheme are labeled in light blue; genes encoding resistance to antimicrobial agents are labeled in red; other genes are labeled in black. B) Venn diagram depicting unique and shared CDSs in each of the *S*. *suis* strains as identified by ortholog analysis. Each strain is represented by one color, and the number of CDSs are displayed in the same color. Numbers in the intersectional regions indicate CDSs shared by two or three strains. Since there may be more than one CDS in the same ortholog cluster, number of CDSs in the intersections are slightly different between strains C) Collinearity of the genomes of *S*. *suis* ST28 strains NSUI002 and 05HAS68. The genomes of the strains were aligned using progressiveMauve. Sequence alignments that are free of rearrangements are shown as colored local collinear blocks (LCBs). Sequence inversions are denoted by differential positioning of the LCBs relative to a reference axis. Several genome rearrangements between NSUI002 and 05HAS68 are noticeable.

We first sequenced to closure the genome of Canadian ST28 strain NSUI002. The genome was a circular chromosome of 2,255,345 bp with a G+C content of 41.1% (Fig 1A). The GC content of the NSUI002 genome was similar to that of the 19 *S*. *suis* genomes previously sequenced to closure (S2 Table). It was also one of the largest *S*. *suis* genomes sequenced so far, and the largest genome of a serotype 2 strain. One reason for the difference in genome sizes between strains is the presence in NSUI002 of one large mobile genetic element (MGE, ~ 83 kbp) spanning from position 1,105,623 to 1,188,671, which carries gene *tetO* encoding resistance to tetracycline, and several other MGE scattered throughout the genome (Fig 1A). We identified 2,221 CDSs in NSUI002. This is a slightly higher number than found in the very recently finished genome of avirulent ST28 Chinese strain 05HAS68 (2,009 CDSs) [13]. Most other closed *S*. *suis* genomes belong to either ST1 or ST7 strains, both included in MLST CC1.

Based on previous reports that questioned the annotation quality of some of the *S*. *suis* closed genomes [39], we chose strain P1/7 as a representative member of CC1 strains to compare the ST28 genomes. Ortholog analysis revealed 1,656 NSUI002 CDSs common between Canadian ST28 strain NSUI002, Chinese ST28 strain O5HAS68 and reference ST1 strain P1/7 (Fig 1B). NSUI002 had 192 unique CDSs, most of them encoded in MGEs. A total of 34 NSUI002 CDSs had an orthologue in P1/7 only. When the Illumina reads for NSUI002 were aligned to the P1/7 reference genome using VAAL, there was a total of 42,896 SNPs between them, as well as 1196 deletions and 738 insertions. Key differences between NSUI002 and P1/7 genomes include the absence in NSUI002 of virulence markers *sly* and *epf* (encoding a hemolysin known as suilysin, and a secreted protein known as extracellular factor, respectively) [40–42]. NSUI002 possessed a *srtG* pilus island which was absent from P1/7 [43].The two strains possessed gene *mrp*, encoding a muramidase-released protein that has been described as important but not essential in virulence [44, 45]. Using the typing scheme developed by Silva *et al* [46], which amplifies a short region of the *mrp* gene, *in silico* PCR identified that strains P1/7 and NSUI002 both possess the 1148 *mrp* variant. However, when comparing the full predicted translated MRP sequences we identified several amino acid differences between the two strains (S1 Fig). In addition, although it has been reported that *mrp* was absent from the genome of Chinese ST28 isolate O5HAS68 [13], we did find this gene in that isolate. When we inspected the reported O5HAS68 genome, *in silico* PCR determined it had *mrp* ^S^ variant (S1 Fig).

Consistent with previous findings in ST28 strains [43], NSUI002 genome had a complete *srtF* pilus cluster, but did not possess pilus cluster *srtBCD*. A truncated *srtE* pilus cluster (lacking genes encoding pilin subunits) was also identified. The genome of strain NSUI002 possessed the same two-component systems (TCSs) and global virulence regulators previously identified in strain 05HAS68 [13]. Namely, NSUI002 contains *ihk/irr*, *ciaRH*, *covR* and *vicK*. Genes encoding other regulators such as *virR*/*virS* and *revS*, present in strain P1/7 [39], were absent from the NSUI002 genome (Table 1). Other regulators such as *salK*/*salR* and *nisK*/*nisR*, so far only found among Chinese ST7 strains [47], were also not identified in the NSUI002 genome. Homology between NSUI002 and ST1 P1/7 CDSs was lower than between NSUI002 and O5HAS68 (Fig 1A and 1B). Both ST28 strains shared a significant number of orthologous CDSs that were not present in P1/7 (Fig 1B). Genome alignments using progressiveMauve [27] identified several areas of genome rearrangements, including inversions (Fig 1C) between the ST28 strains. The majority of these rearrangements occurred at genome areas encoding transposases (76.7%). Other genomic rearrangements occurred at rRNA operons, or sites encoding phage integrases and/or phage related proteins. Finally, we identified that while both ST28 strains possessed gene *tetO*, encoding resistance to tetracycline, only the Canadian ST28 strain NSUI002 possessed gene *ermB*, encoding resistance to macrolides. Resistance to tetracycline and to macrolide and glycosamides is carried in different MGE inserted in different regions of the NSUI002 genome (Fig 1A and S2 Fig).

**Table 1:** Presence of two component or standalone global regulators in the different ST28 and ST1 strains.

Regulator	NSUI002 (ST28)	05HAS68 (ST28)	P1/7 (ST1)
*ihK*/*irr*	+	+	+
*ciaRH*	+	+	+
*vicK*	+	+	+
*salK*/*salR*	-	-	-
*nisK*/*nisR*	-	-	-
*virR*/*virS*	-	-	+
*covR*	+	+	+
*revS*	-	-	+

### Sequencing of additional ST28 strains and presence of markers of antimicrobial resistance

We next sequenced the genomes of 50 additional ST28 serotype 2 strains using Illumina technology. The number of short reads obtained for each strain and the calculated coverage are presented in S1 Table. ST28 was confirmed in all 50 strains by extracting MLST information directly from the short-read WGS data using SRST2 [21]. We also used SRST2 to identify genes associated with antimicrobial resistance in our strain collection. This information is important to obtain, as *S*. *suis* is a microorganism that can live in different animal hosts as well as the human host and thus there is potential for possible intersections between animal and human resistomes [48, 49]. Gene *tetO* was identified in 49 of the strains, while gene *ermB* was present in 41. Gene *lnuC*, associated with clindamycin and lincomycin resistance, and genes *ant6* and *aph3*’, associated with resistance to aminoglycosides, were identified in 8 and 2 of the strains, respectively (S1 Table). Resistance to these antimicrobials has previously been identified in several other diverse *S*. *suis* isolates [48]. We did not notice any clear indication of geographical differences in antimicrobial resistance markers among the strains in this study.

### Complex population structure of ST28 *S. suis* revealed by phylogenetic analysis

We next defined the ST28 *S*. *suis* core and pan genomes by performing ortholog analysis between the reference NSUI002 genome and annotated pseudochromosomes of all other 50 ST28 strains. We identified 1,786 core gene clusters and 2,776 pan genome clusters (S3 Fig). Previously, Zhang *et al*. studied 13 strains of *S*. *suis* belonging to seven different serotypes and at least six different STs, and defined a core genome size of 1,343 genes and a pan genome of 3,585 genes [50]. Since these authors analyzed strains of highly diverse genetic backgrounds, a larger core genome and smaller pan genome was expected in our cohort. We also identified a total of 31,488 non-redundant SNPs between all strains and the NSUI002 reference. To establish phylogenies, we defined a reduced ST28 core genome by first eliminating from the analysis gene clusters encoded in MGEs, and those CDSs present in all strains but whose length differed by > 9bp among the isolates. This left a final number of 1422 core gene clusters (1,269,771 bp) between the 51 ST28 *S*. *suis* strains under investigation. In this reduced core genome, 11,305 SNPs were identified. However, most of these SNPs were clustered in a few discrete areas of the core genome, which is suggestive of recombination.

**Fig 2:**
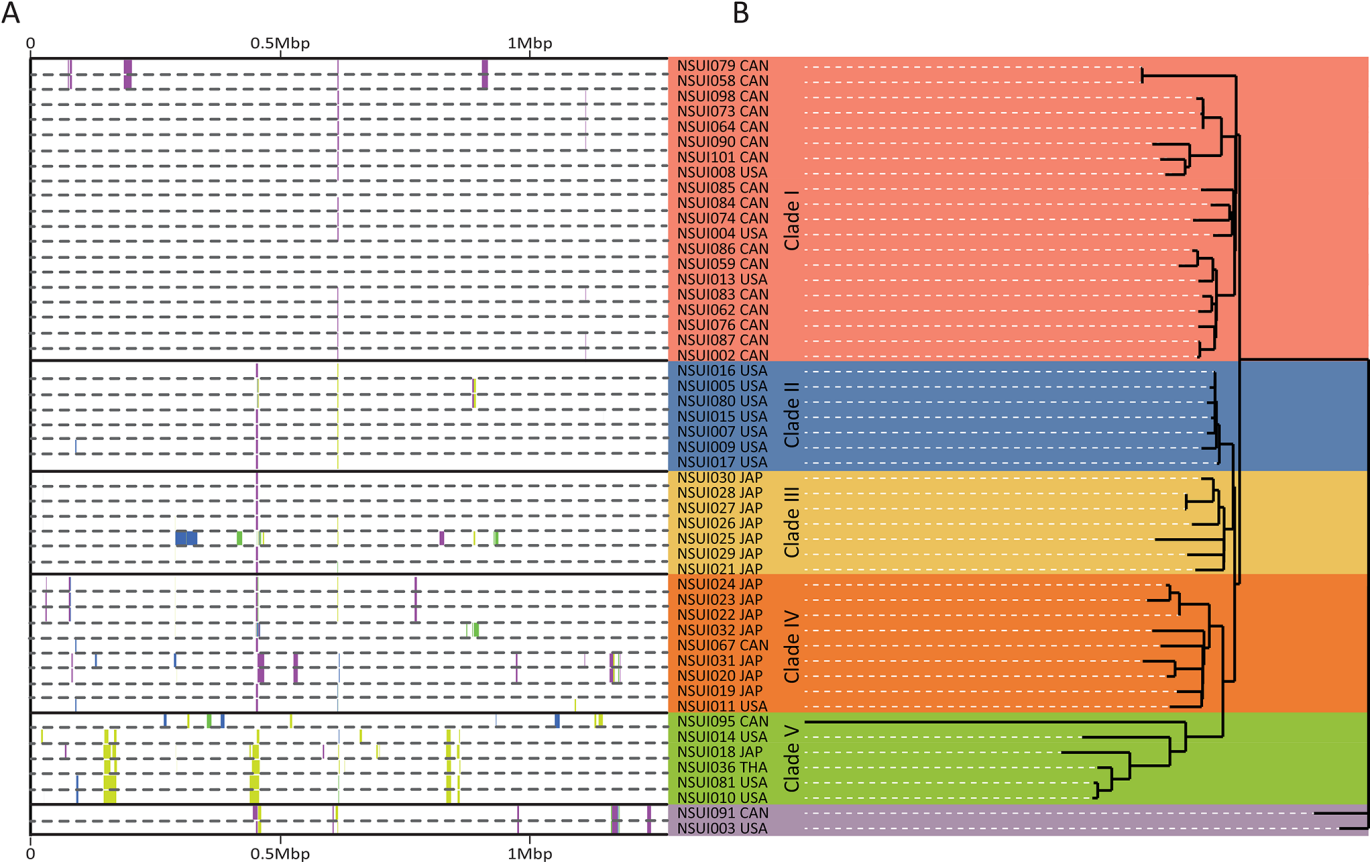
A) Results of Bayesian analysis of recombination for the 51 ST28 *S*. *suis* strains. The names and countries of isolation of the strains are shown on the right. The colored bars denote the recombination events in the strains along the core genome. The coloring of the bars at a specific genomic location reflects the clustering of the recombination events into groups, and is unrelated to other bars at distant genomic locations. CAN: Canada, USA: United States of America; JAP: Japan; Tha: Thailand. B) Neighbor-joining phylogenetic tree depicting the relationships between the 51 ST28 *S*. *suis* strains. The tree was constructed using 1,421 SNPs identified against the core genome (see text for details). Two singletons and five distinct clades (I to V) were identified.

Recombination is common among some streptococcal species for which extensive genetic recombination within populations has been observed [51–53]. Extensive recombination among highly diverse *S*. *suis* isolates has also very recently been described [54]. To assess recombination in more detail, we used Bayesian analysis, which revealed 49 regions of recombination containing 441 genes (Fig 2A and S3 Table). Neighbor joining phylogenetic analysis using the 1,421 informative SNPs remaining after exclusion of areas of the core genome having undergone recombination revealed two singletons (NSUI091, from Canada, and NSUI003 isolated in the USA), and two larger clades (Fig 2B). One of them, identified here as clade I, comprised most of the Canadian strains in our collection, including the NSUI002 reference strain, and three US strains. The second major clade could be divided into four different subclades, identified here as clades II-V.

**Fig 3:**
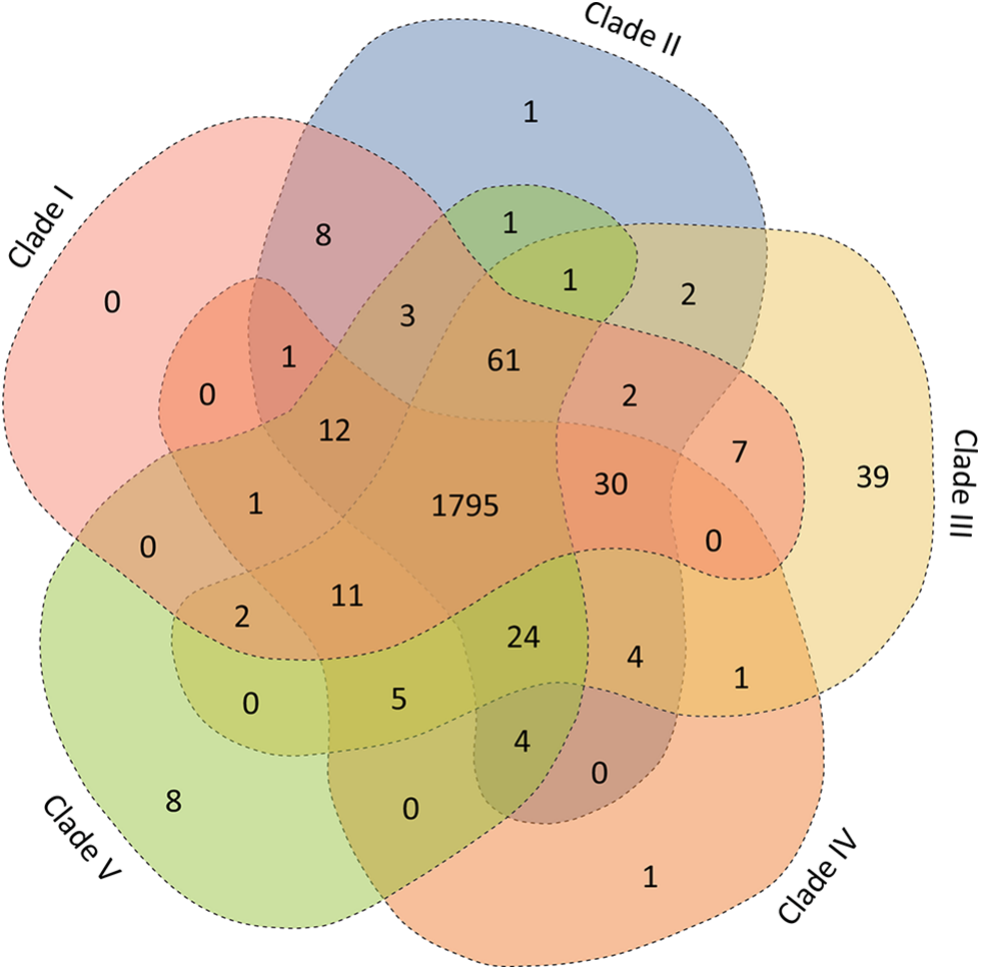
Venn diagram depicting unique and shared ortholog gene clusters in each of the five clades defined among the *S*. *suis* strains. Numbers shown in the different sections indicate the numbers of ortholog groups. The two ST28 singletons were not included in this analysis.

Clades II and III had a strong signal of geographical structure: clade II contained solely US isolates, while clade III was formed solely by strains isolated in Japan. Interestingly, while most strains found in clade IV were isolated in Japan, one Canadian and one US strain were also found in this clade. Import into Japan of live pigs from either the US or Canada for the purposes of breeding occurs frequently. Thus, we hypothesize that clade IV may have originated from *S*. *suis* ST28 strains that were introduced to Japan by import of live hogs from North America. Similarly, clade V had no unambiguous signal of geographic clustering and was formed by three US, one Canadian, one Japanese, and one Thai strain, the latter isolated from a case of human disease (Fig 2B). We next compared gene content among the five clades defined by phylogenetic analysis. The total number of common genes was 1795 (Fig 3). As a group, clade I strains did not possess unique gene content, while one unique gene cluster was found among strains of each clades II and IV. A total of 39 genes clusters were specific of clade III strains. Finally, clade V strains had 8 unique gene clusters. S4–S8 Tables list genes found in all strains of each clade.

### Significant differences in virulence between ST28 *S. suis* strains of clades I and V

**Fig 4:**
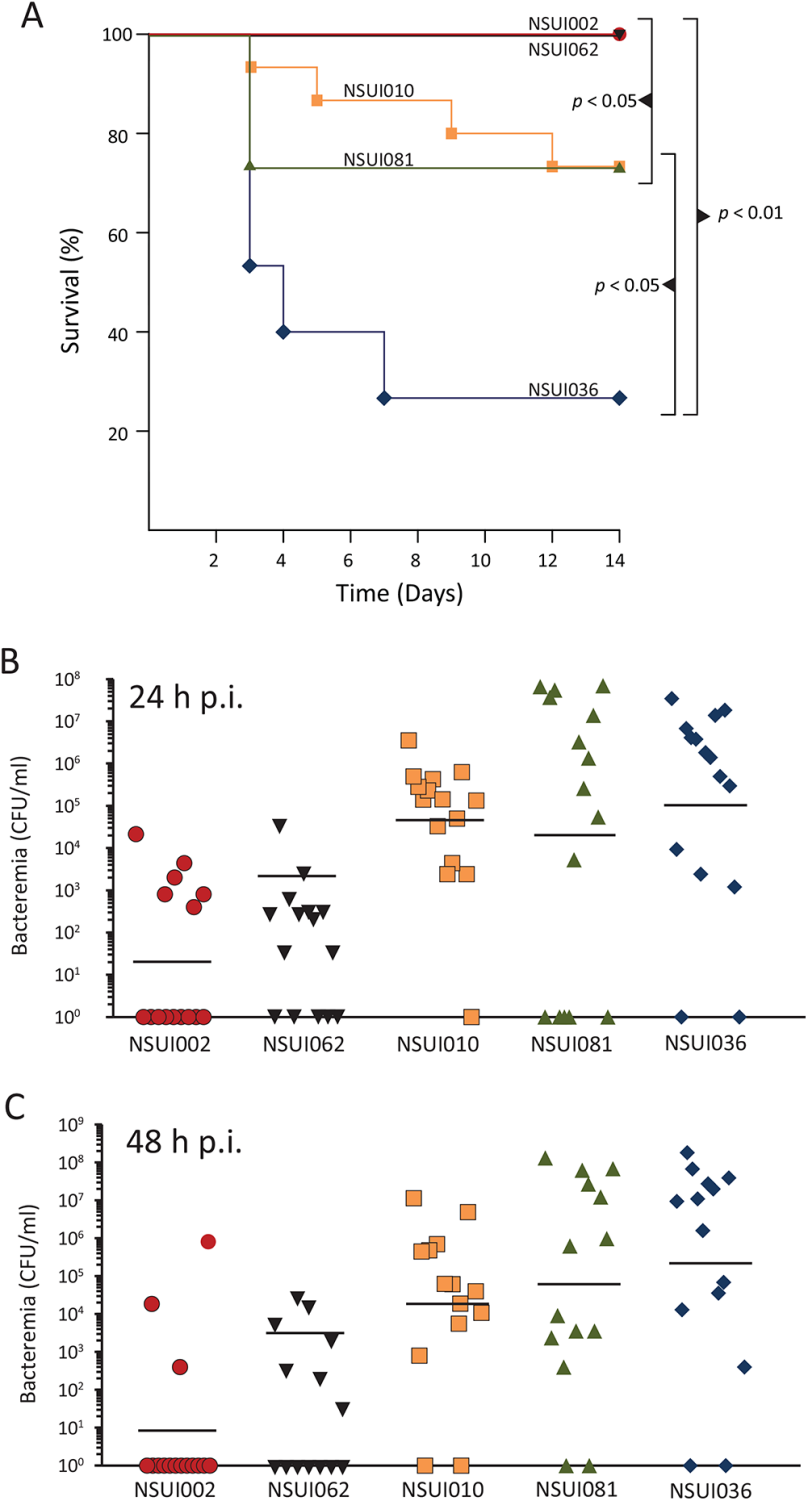
Results of animal experimental infections. **A)** Survival of mice inoculated with the different *S*. *suis* ST28 strains. All the mice in the NSUI002 and NSUI062 groups survived, while approx. 75% of the animals in the NSUI036 group died from septicemia or meningitis. Animals that received strains NSUI010 or NSUI081 showed reduced mortality compared to NSUI036. Significant differences in survival (LogRank test) are depicted in the Fig **Bacteremia at 24 h (B) and 48 h (C) post-infection (pi)**. NSUI002 and NSUI062 were isolated at lower titers than the other three strains following inoculation. The different symbols represent values from individual mice. The horizontal lines indicate the geometrical mean for each group. Significant differences in isolation from blood were noted at 24 h between NSUI036 and NSUI002 and NSUI062 and 48 h pi between NSUI002 and NSUI010, NSUI081 and NSUI036 and between NSUI062 and NSUI036 only (ANOVA on ranks, P < 0.05).

Previous studies that each analyzed one ST28 strain have led to the notion that ST28 *S*. *suis* are typically avirulent or of low virulence [11–13]. However, this notion can be challenged based on reports describing frequent isolation of ST28 strains from diseased pigs in some countries, as well as four human cases of *S*. *suis* ST28 disease [14–19]. Inasmuch as our genomics and phylogenetic analysis revealed that rather than being a homogeneous group of organisms, ST28 strains are genetically heterogeneous, we hypothesized that these genetic differences may, in some cases, correlate with dissimilar virulence potential. To begin to test this hypothesis we compared the virulence of two selected clade I strains (NSUI002 and NSUI062) and three selected clade V strains (NSUI036, NSUI081 and NSUI010) in a murine model of infection. We chose clade I because previous results had demonstrated low virulence of strain NSUI002 in a murine infection model [11]; clade V strains were selected because there was no obvious geographic clustering structure and because this clade included a human isolate. Although swine is *S*. *suis* natural host, mice have frequently been used as a model to study the pathogenesis of *S*. *suis* diseases. Indeed, several reliable murine models using different mouse strains and routes of infection have been validated for *S*. *suis* [36, 55–57]. Here we used one of these models that uses C57BL/6 mice and the intraperitoneal route of infection [36]. Consistent with previous findings [11], no mice in the NSUI002 group died (Fig 4A) nor showed clinical signs associated with *S*. *suis* infection, with the exception of slight depression following inoculation which subsided 24 h pi. Bacteria could not be isolated from the blood of most mice in this group at 24 h pi (Fig 4B). Similar results were observed in the group that received clade I strain NSUI0062, although bacteremia was observed in more animals at 24 and at 48 h pi (Fig 4B and 4C) in this group than in the NSUI002 group. In strong contrast, mice that received clade V strain NSUI036 showed severe clinical signs associated with septicemia, such as depression, swollen eyes, weakness, and prostration during the first 24 h pi. In fact, several mice died or met standard criteria for euthanasia during the first 4 days pi. There were several cases of meningitis between day 4 and day 6 pi in this group. *S*. *suis* was isolated in pure cultures at high titers (> 1 x 10^7^ CFU/ml in some animals) from blood samples in the NSUI036 group (Fig 4B and 4C). The other clade V strains evaluated here (NSUI010 and NSUI081) also caused relatively severe clinical signs and induced high bacteremia in inoculated mice (Fig 4B and 4C). Although mortality was lower than in the NSUI036 group (Fig 4A), statistical analysis revealed significant differences in survival between NSUI002 and the two clade I strains.

**Fig 5:**
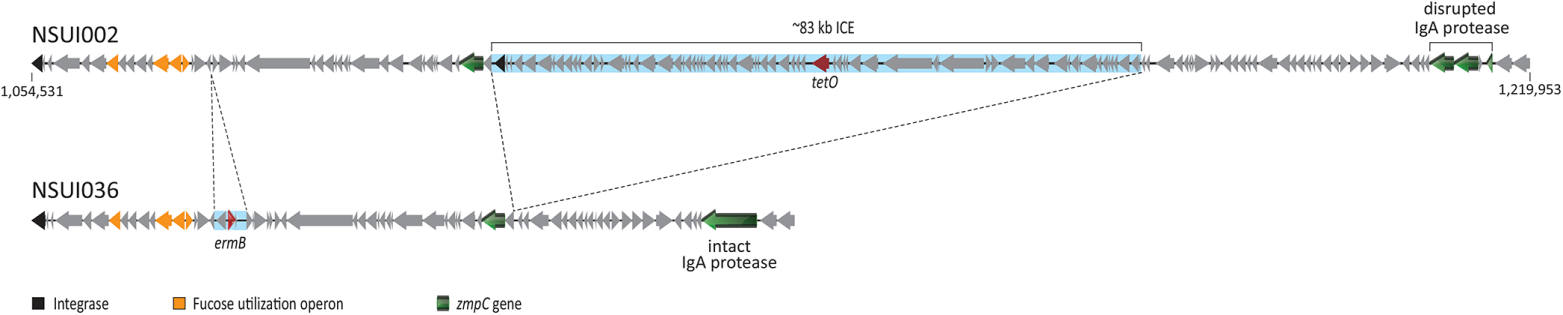
Genetic organization and predicted open reading frames of NSUI002 (clade I) and NSUI036 (clade V) regions containing a fucose utilization operon and *zmpC* genes. The region spans from position 1,054,531 to 1,219,953 in strain NSUI002, and contains 152 CDSs. In NSUI036, the region is notably smaller (83,658 bp). Differences in size are mainly due to the absence in the genome of strain NSUI036 of an approx. 83 kbp mobile genetic element (MGE), highlighted in light blue, that contains gene *tetO*. Other differences include a small MGE (highlighted in light blue) in NUI036 that contains gene *ermB*. In the conserved area, we observed a high degree of gene content conservation, with the exception of a *zmpC* gene also known as *iga*, encoding an IgA protease involved in *S*. *suis* virulence [59, 60], which is intact in NSUI036 but disrupted by a transposon insertion in strain NSUI002.

Inspection of unique gene content in clade V strains identified two genes encoding an ABC-type cobalt transport system. This ABC transporter has previously been found to be upregulated *in vivo* by virulent *S*. *suis* strains [58]. Another key difference between clade V and clade I strains was that in the latter group a gene encoding a zinc-dependent IgA protease previously found to be important in *S*. *suis* virulence [59, 60] was disrupted by a transposon insertion, while the gene was intact in clade V strains (Fig 5). Interestingly, in clade I strains, an ICE carrying *tetO*, which is absent from clade V strains, lies between another gene encoding a different putative zinc-dependent protease present in strains of both clades (Fig 5). We also discovered that NSUI036, the most virulent clade V strain, and the only human case included in our collection, had a 1bp insertion in the *sgp2* gene predicted to result in premature termination of translation of Sgp2, the putative adhesin of the *srtG* pilus [61]. Previous reports in *Streptococcus pyogenes* have shown that strains impaired in pilus production are better fit to survive in blood and cause invasive disease [62].

## Concluding Remarks

Recent technological advances in whole-genome sequencing now permit the cost-effective and rapid generation of data that can be used to precisely inform us about the population structure of pathogenic or commensal bacteria [63]. The characteristics of *S*. *suis* serotype 2 strains belonging to ST28 (highly prevalent in North America) are poorly known. The use of whole-genome sequencing allowed us to uncover a relatively high level of genetic diversity among a large collection of strains isolated from diseased pigs and humans in different geographies. Experimental animal infections also discovered significant differences in virulence among strains belonging to two of the five different clades identified by whole-genome SNP-based phylogenetic analysis. Our results clearly highlight the limitations of typing *S*. *suis* strains using the commonly used MLST scheme [10], which failed to reveal the genetically heterogeneous nature of our strain collection. Furthermore, it now seems apparent that using MLST alone as a predictor of *S*. *suis* strain virulence can be misleading. Indeed, previous reports have proposed that ST28 *S*. *suis* strains are of low virulence [7, 11, 13], while here we show that at least some ST28 serotype 2 strains can induce severe disease in an experimental infection model. A key difference between these previous studies and this work is that, while the former drew their conclusions from results obtained after evaluation of the virulence of a single ST28 strain, here we used a population-based strain collection. In this regard, our results are consistent with previous findings describing frequent isolation of ST28 strains from diseased swine, and from human cases in China, Japan and Thailand [14–18]. Our work is the first step towards better characterization of this diverse group of organisms heretofore considered genetically homogeneous. Further mining of the genome data generated in this study, coupled with mutagenesis of selected virulence factor candidates and animal studies will be instrumental in understanding the genetic basis of virulence differences among serotype 2 ST28 *S*. *suis* strains.

## Supporting Information

S1 Fig ClustalW alignment of the predicted translated sequences of the different *mrp* gene variants of ST28 strains 05HAS68 and NSUI002, and ST1 strain P1/7. (PDF)

S2 Fig Diagram showing the genetic organization of mobile genetic elements carrying genes encoding resistance to antimicrobial agents in ST28 strains NSUI002 and 05HAS68. (PDF)

S3 Fig Core and pan-genome of the 51 ST 28 *S*. *suis* strains. (PDF)

S1 Table *Streptococcus suis* strains used in this study. (PDF)

S2 Table Characteristics of the NSUI002 and other previously closed *Streptococcus suis* genomes. (PDF)

S3 Table Recombination among the 51 ST28 *S*. *suis* strains as defined by BratNextGen. (PDF)

S4 Table Common ortholog gene clusters among clade I ST28 *Streptococcus suis* strains. (PDF)

S5 Table Common ortholog gene clusters among clade II ST28 *Streptococcus suis* strains. (PDF)

S6 Table Common ortholog gene clusters among clade III ST28 *Streptococcus suis* strains. (PDF)

S7 Table Common ortholog gene clusters among clade IV ST28 *Streptococcus suis* strains. (PDF)

S8 Table Common ortholog gene clusters among clade V ST28 *Streptococcus suis* strains. (PDF)
