# Supplementary material for: Complex Population Structure and Virulence Differences among Serotype 2 Streptococcus suis Strains Belonging to Sequence Type 28
Source: PLoS One. 2015 Sep 16;10(9):e0137760. doi: 10.1371/journal.pone.0137760 (PMC4574206; doi:10.1371/journal.pone.0137760)
Supplement: S2 Fig — (PDF) [file pone.0137760.s002.pdf]

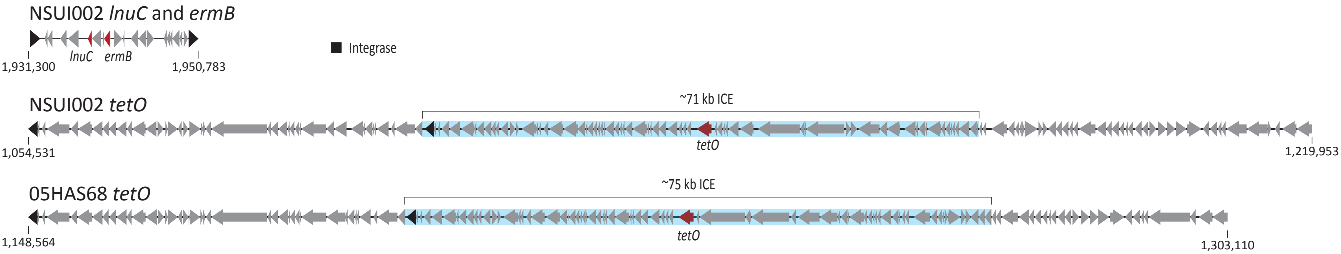

S2 Fig. Diagram showing the genetic organization of mobile genetic elements carrying genes encoding resistance to antimicrobial agents in ST28 strains NSUI002 and 05HAS68. Positions relative to the corresponding genomes are provided.
