# Supplementary material for: Complex Population Structure and Virulence Differences among Serotype 2 Streptococcus suis Strains Belonging to Sequence Type 28
Source: PLoS One. 2015 Sep 16;10(9):e0137760. doi: 10.1371/journal.pone.0137760 (PMC4574206; doi:10.1371/journal.pone.0137760)
Supplement: S3 Fig — (PDF) [file pone.0137760.s003.pdf]

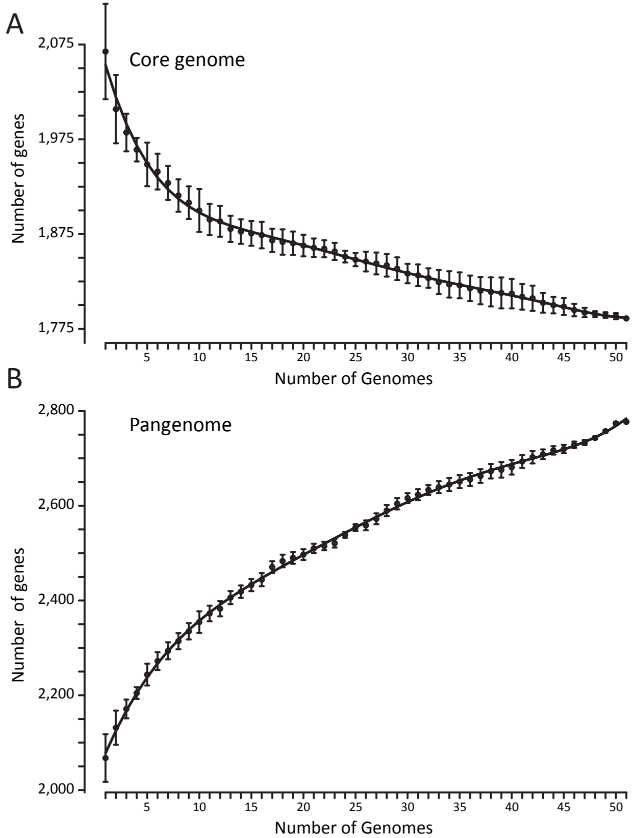

S3 Fig. Core and pan-genome of the 51 ST 28 *S. suis* strains. A) The trend line for the core genome follows the hexic function $E(y|x)=2.093 \times 10^3 - 44.84x + 4.051x^2 - 0.1997x^3 + 5.294 \times 10^{-3}x^4 - 7.155 \times 10^{-5}x^5 + 3.871 \times 10^{-7}x^6$, and has an R^2 value of 0.9402. The use of all 6 powers of x were significant ($p < 0.01$). B) The trend line for the pan genome follows the quintic function $E(y|x)=2.024 \times 10^3 + 57x - 3.45x^2 + 0.1308x^3 - 2.436 \times 10^{-3}x^4 + 1.725 \times 10^{-5}x^5$, and has an R^2 value of 0.9661. The use of all 5 powers of x were highly significant ($p < 0.001$).
