# Supplementary material for: Complex Population Structure and Virulence Differences among Serotype 2 Streptococcus suis Strains Belonging to Sequence Type 28
Source: PLoS One. 2015 Sep 16;10(9):e0137760. doi: 10.1371/journal.pone.0137760 (PMC4574206; doi:10.1371/journal.pone.0137760)
Supplement: S1 Table — (PDF) [file pone.0137760.s004.pdf]

S1 Table. *Streptococcus suis* strains used in this study.

Strain	Country	Host	Isolation date	Illumina sequencing statistics		Presence of antimicrobial resistance genes [1]					
				Nº of reads	Coverage [2]	*ant(6)-Ia*	*aph(3')-III*	*erm(B)*	*lnu(B)*	*lnu(C)*	*tet(O)*
NSUI002	Canada	Swine	05/2008	3,071,172	204.26	-	-	+	-	+	+
NSUI003	USA	Swine	NA [3]	1,176,762	52.70	-	-	+	-	-	+
NSUI004	USA	Swine	01/2004	3,036,440	135.98	-	-	+	-	-	+
NSUI005	USA	Swine	12/2004	2,711,158	121.41	-	-	+	-	-	+
NSUI007	USA	Swine	NA	1,756,994	78.68	-	-	+	-	-	+
NSUI008	USA	Swine	NA	1,450,038	64.94	-	-	+	-	-	+
NSUI009	USA	Swine	01/2004	4,842,954	216.88	-	-	+	-	-	+
NSUI010	USA	Swine	12/2003	3,759,796	168.37	-	-	+	-	-	+
NSUI011	USA	Swine	05/2005	1,944,814	87.09	-	-	+	-	-	+
NSUI013	USA	Swine	05/2005	2,110,588	94.52	-	-	+	-	-	+
NSUI014	USA	Swine	05/2005	2,423,066	108.51	-	-	+	-	-	+
NSUI015	USA	Swine	06/2005	1,828,742	121.63	-	-	+	-	-	+
NSUI016	USA	Swine	06/2005	2,133,192	141.88	-	-	+	-	-	+

Strain	Country	Host	Isolation date	Illumina sequencing statistics		Presence of antimicrobial resistance genes [1]					
				Nº of reads	Coverage [2]	*ant(6)-Ia*	*aph(3')-III*	*erm(B)*	*lnu(B)*	*lnu(C)*	*tet(O)*
NSUI017	USA	Swine	NA	3,381,426	224.89	-	-	+	-	-	+
NSUI018	Japan	Swine	12/2005	2,966,222	197.28	-	-	+	-	-	+
NSUI019	Japan	Swine	01/1990	2,851,096	127.68	-	-	+	-	-	+
NSUI020	Japan	Swine	01/1995	2,691,186	120.52	-	-	+	-	-	+
NSUI021	Japan	Swine	04/1996	2,295,282	102.79	-	-	+	-	-	+
NSUI022	Japan	Swine	06/1991	1,342,564	60.12	-	-	+	-	-	+
NSUI023	Japan	Swine	08/1993	2,655,526	118.92	-	-	+	-	-	+
NSUI024	Japan	Swine	07/1996	2,740,988	122.75	-	-	+	-	-	+
NSUI025	Japan	Swine	NA	2,168,578	97.11	-	-	-	-	-	+
NSUI026	Japan	Swine	NA	3,624,700	162.32	-	-	+	-	-	+
NSUI027	Japan	Swine	11/1992	3,736,496	167.33	-	-	+	-	-	+
NSUI028	Japan	Swine	12/1992	3,556,094	159.25	-	-	+	-	-	+
NSUI029	Japan	Swine	NA	2,235,898	100.13	-	-	-	-	-	-
NSUI030	Japan	Swine	03/1994	3,806,082	170.45	-	-	+	-	-	+
NSUI031	Japan	Swine	NA	3,177,256	142.29	-	-	+	-	-	+

Strain	Country	Host	Isolation date	Illumina sequencing statistics		Presence of antimicrobial resistance genes [1]					
				Nº of reads	Coverage [2]	*ant(6)-Ia*	*aph(3')-III*	*erm(B)*	*lnu(B)*	*lnu(C)*	*tet(O)*
NSUI032	Japan	Swine	NA[3]	1,575,696	70.56	-	-	-	-	-	-
NSUI036	Thailand	Human	06/2002	2,521,586	112.92	-	-	+	-	-	+
NSUI058	Canada	Swine	04/2008	5,621,746	251.76	-	-	-	-	-	+
NSUI059	Canada	Swine	04/2008	4,188,098	187.55	-	-	+	-	-	+
NSUI062	Canada	Swine	07/2007	5,191,654	232.50	-	-	-	-	-	+
NSUI064	Canada	Swine	09/2007	4,382,760	196.27	-	-	+	+	-	+
NSUI067	Canada	Swine	April-2008	5,433,840	243.34	-	-	+	-	-	+
NSUI073	Canada	Swine	May-2008	5,293,456	237.05	-	-	+	+	-	+
NSUI074	Canada	Swine	June-2008	3,345,194	149.81	-	-	-	-	-	+
NSUI076	Canada	Swine	June-2008	5,098,552	228.33	-	-	-	-	-	+
NSUI079	Canada	Swine	June-2008	2,336,800	104.65	-	-	-	-	-	+
NSUI080	USA	Swine	10/2004	3,796,506	170.02	-	-	+	-	-	+
NSUI081	USA	Swine	NA	4,892,548	219.10	-	-	+	-	-	+
NSUI083	Canada	Swine	03/2010	4,062,304	181.92	-	-	-	+	-	+
NSUI084	Canada	Swine	05/2010	5,284,400	236.65	-	-	-	-	-	+

Strain	Country	Host	Isolation date	Illumina sequencing statistics		Presence of antimicrobial resistance genes [1]					
				Nº of reads	Coverage [2]	*ant(6)-Ia*	*aph(3')-III*	*erm(B)*	*lnu(B)*	*lnu(C)*	*tet(O)*
NSUI085	Canada	Swine	06/2010	7,504,846	336.09	-	-	+	-	-	+
NSUI086	Canada	Swine	02/2010	9,555,692	427.93	-	-	+	-	-	+
NSUI087	Canada	Swine	04/2011	9,391,748	420.59	-	-	+	-	+	+
NSUI090	Canada	Swine	09/2008	1,439,108	95.71	-	-	+	+	-	+
NSUI091	Canada	Swine	09/2008	2,328,408	154.86	+	+	+	+	-	+
NSUI095	Canada	Swine	05/2008	6,856,094	307.03	+	+	+	-	-	+
NSUI098	Canada	Swine	06/2008	5,525,568	247.45	-	-	+	+	-	+
NSUI101	Canada	Swine	10/2007	1,558,798	69.81	-	-	+	-	-	+

[1] As determined using SRST2 (Inouye *et al*. SRST2: Rapid genomic surveillance for public health and hospital microbiology labs. Genome Med. 2014;6(11):90) from whole-genome short-read data.

[2] Based on an average genome size of 2,255,345 bp.

[3] NA: Not available.
