# Supplementary material for: Complex Population Structure and Virulence Differences among Serotype 2 Streptococcus suis Strains Belonging to Sequence Type 28
Source: PLoS One. 2015 Sep 16;10(9):e0137760. doi: 10.1371/journal.pone.0137760 (PMC4574206; doi:10.1371/journal.pone.0137760)
Supplement: S2 Table — (PDF) [file pone.0137760.s005.pdf]

S2 Table: Characteristics of the NSUI002 and other previously closed *Streptococcus suis* genomes.

Strain	Serotype	Sequence Type	Length	% GC content	Nº of CDSs	GenBank Acc. Nº
NSUI002	2	28	2,255,345	41.10	2221	CP011419
ST3	3	35	2,028,815	41.30	1862	NC_015433.1
TL13	16	Novel[1]	2,038,146	41.30	1874	NC_021213.1
SS12	1/2	Novel; 1-like[2]	2,096,866	41.20	1989	NC_017619.1
JS14	14	Novel; 7-like[3]	2,137,435	41.20	1979	NC_017618.1
BM407	2	1	2,146,229	41.10	1947	NC_012926.1
P1/7	2	1	2,007,491	41.30	1898	NC_012925.1
S735	2	1	1,980,887	41.40	1840	NC_018526.1
SC84	2	7	2,095,898	41.10	1973	NC_012924.1
D9	7	29	2,177,656	41.00	1966	NC_017620.1
YB51	3	35	2,043,655	41.30	1869	NC_022516.1
T15	2	19	2,240,234	41.00	2100	NC_022665.1
GZ1	2	1	2,038,034	41.40	1872	NC_017617.1
ST1	1	13	2,034,321	41.40	1869	NC_017950.1
A7	2	Novel; 7-like[3]	2,038,409	41.20	1891	NC_017622.1
D12	9	619	2,183,059	41.30	2008	NC_017621.1
05HAS68	2	28	2,188,363	41.20	2065	NZ_CP002007.1
SC070731	ND[4]	Novel; 7-like[3]	2,138,568	41.20	2003	NC_020526.1
98HAH33	2	Novel; 7-like or 377-like[5]	2,095,698	41.10	2185	CP000408.1
05ZYH33	2	Novel; 7-like or 377-like[5]	2,096,309	41.10	2186	NC_009442.1
6407	4	54	2,292,360	41.00	2127	NZ_CP008921.1

[1] Traditional MLST has not been used to type this strain. We determined MLST alleles from the available genome sequencing data. The particular allele combination for this strain was *aroA* 15; *cpn60* 58; *dpr* 42; *gki* 4; *mutS* 16; *recA* 16; *thrA* 12. Thus, the strain is a single-locus variant of ST163, ST172, ST192 and ST477.

[2] Traditional MLST has not been used to type this strain. We determined MLST alleles from the available genome sequencing data. The particular allele combination for this strain was *aroA* 1; *cpn60* 162; *dpr* 1; *gki* 1; *mutS* 1; *recA* 1; *thrA* 1. Thus, the strain is a single-locus variant of ST1.

[3] Traditional MLST has not been used to type this strain. We determined MLST alleles from the available genome sequencing data. The particular allele combination for this strain was *aroA* 1; *cpn60* 162; *dpr* 1; *gki* 1; *mutS* 1; *recA* 1; *thrA* 3. Thus, the strain is a single-locus variant of ST7.

[4] Not determined

[5] Traditional MLST has not been used to type this strain. We determined MLST alleles from the available genome sequencing data. The particular allele combination for this strain was *aroA* 177; *cpn60* 162; *dpr* 1; *gki* 1; *mutS* 1; *recA* 1; *thrA* 3. Thus, the strain is a 2-loci variant of ST377 and ST7.
