# Supplementary material for: Complex Population Structure and Virulence Differences among Serotype 2 Streptococcus suis Strains Belonging to Sequence Type 28
Source: PLoS One. 2015 Sep 16;10(9):e0137760. doi: 10.1371/journal.pone.0137760 (PMC4574206; doi:10.1371/journal.pone.0137760)
Supplement: S3 Table — (PDF) [file pone.0137760.s006.pdf]

S3 Table. Recombination among the 51 ST28 *S. suis* strains as defined by BratNextGen (Martinnen *et al*. Bayesian modeling of recombination events in bacterial populations. BMC Bioinformatics. 2008;9:421).

NSUI002 Gene Locus ID	Gene Position in Core Genome	Gene Position NSUI002 Genome	Strains Showing Recombination
NSUI002_00044	18906..22625	31986..35705	NSUI014
NSUI002_00045	22626..24080	35708..37162	NSUI014
NSUI002_00055	30471..31556	45738..46823	NSUI022, NSUI023, NSUI024
NSUI002_00056	31557..32480	46810..47733	NSUI022, NSUI023, NSUI024
NSUI002_00057	32481..33773	47768..49060	NSUI022, NSUI023, NSUI024
NSUI002_00058	33774..34598	49572..50396	NSUI022, NSUI023, NSUI024
NSUI002_00144	68592..68801	108492..108701	NSUI018
NSUI002_00145	68802..69179	108994..109371	NSUI018
NSUI002_00146	69180..70436	109429..110685	NSUI018
NSUI002_00148	70437..74009	113819..117391	NSUI018
NSUI002_00153	76278..77243	123735..124700	NSUI058, NSUI079
NSUI002_00154	77244..77525	124702..124983	NSUI058, NSUI079
NSUI002_00155	77526..77933	124964..125371	NSUI058, NSUI079
NSUI002_00156	77934..78188	125382..125636	NSUI022, NSUI023, NSUI024
NSUI002_00157	78189..78623	125623..126057	NSUI022, NSUI023, NSUI024
NSUI002_00159	78624..79577	126502..127455	NSUI022, NSUI023, NSUI024
NSUI002_00160	79578..80765	127505..128692	NSUI020, NSUI022, NSUI023, NSUI024, NSUI031, NSUI058, NSUI079
NSUI002_00162	80766..82022	129614..130870	NSUI020, NSUI022, NSUI023, NSUI024, NSUI031, NSUI058, NSUI079
NSUI002_00163	82023..83084	130916..131977	NSUI020, NSUI031, NSUI058, NSUI079
NSUI002_00164	83085..83369	132190..132474	NSUI058, NSUI079
NSUI002_00165	83370..83690	132471..132791	NSUI058, NSUI079
NSUI002_00167	83691..84314	133811..134434	NSUI058, NSUI079

NSUI002 Gene Locus ID	Gene Position in Core Genome	Gene Position NSUI002 Genome	Strains Showing Recombination
NSUI002_00177	89829..90839	145164..146174	NSUI009, NSUI010, NSUI011, NSUI067, NSUI081
NSUI002_00178	90840..92039	146434..147633	NSUI009, NSUI010, NSUI011, NSUI067, NSUI081
NSUI002_00179	92040..92552	148311..148823	NSUI009, NSUI010, NSUI011, NSUI067, NSUI081
NSUI002_00180	92553..92924	148903..149274	NSUI009, NSUI010, NSUI011, NSUI067, NSUI081
NSUI002_00181	92925..94271	149301..150647	NSUI009, NSUI010, NSUI011, NSUI067, NSUI081
NSUI002_00182	94272..95951	150870..152549	NSUI010, NSUI081
NSUI002_00234	125127..127616	210949..213438	NSUI031
NSUI002_00235	127617..128246	213502..214131	NSUI031
NSUI002_00236	128247..129137	214141..215031	NSUI031
NSUI002_00237	129138..130121	215116..216099	NSUI031
NSUI002_00239	130122..131747	218036..219661	NSUI031
NSUI002_00271	144516..145850	245391..246725	NSUI010, NSUI014, NSUI018, NSUI036, NSUI081
NSUI002_00287	145851..146699	257902..258750	NSUI010, NSUI014, NSUI018, NSUI036, NSUI081
NSUI002_00288	146700..147287	258761..259348	NSUI010, NSUI014, NSUI018, NSUI036, NSUI081
NSUI002_00289	147288..148634	259583..260929	NSUI010, NSUI014, NSUI018, NSUI036, NSUI081
NSUI002_00290	148635..149573	261152..262090	NSUI010, NSUI014, NSUI018, NSUI036, NSUI081
NSUI002_00291	149574..150806	262423..263655	NSUI010, NSUI014, NSUI018, NSUI036, NSUI081
NSUI002_00292	150807..151634	263667..264494	NSUI010, NSUI014, NSUI018, NSUI036, NSUI081
NSUI002_00293	151635..152417	264544..265326	NSUI010, NSUI014, NSUI018,

NSUI002 Gene Locus ID	Gene Position in Core Genome	Gene Position NSUI002 Genome	Strains Showing Recombination
			NSUI036, NSUI081
NSUI002_00294	152418..153197	265328..266107	NSUI010, NSUI014, NSUI018, NSUI036, NSUI081
NSUI002_00295	153198..154181	266100..267083	NSUI010, NSUI014, NSUI018, NSUI036, NSUI081
NSUI002_00296	154182..154715	267168..267701	NSUI010, NSUI014, NSUI018, NSUI036, NSUI081
NSUI002_00297	154716..155507	267827..268618	NSUI010, NSUI018, NSUI036, NSUI081
NSUI002_00299	155508..157196	269496..271184	NSUI010, NSUI018, NSUI036, NSUI081
NSUI002_00300	157197..158255	271236..272294	NSUI010, NSUI018, NSUI036, NSUI081
NSUI002_00301	158256..159011	272291..273046	NSUI010, NSUI018, NSUI081
NSUI002_00302	159012..159338	273033..273359	NSUI010, NSUI018, NSUI081
NSUI002_00303	159339..159719	273668..274048	NSUI010, NSUI018, NSUI081
NSUI002_00304	159720..160415	274041..274736	NSUI010, NSUI081
NSUI002_00305	160416..160988	274770..275342	NSUI010, NSUI081
NSUI002_00306	160989..161834	275616..276461	NSUI010, NSUI081
NSUI002_00307	161835..163367	276590..278122	NSUI010, NSUI014, NSUI081
NSUI002_00308	163368..163976	278234..278842	NSUI010, NSUI014, NSUI081
NSUI002_00311	163977..164123	283576..283722	NSUI010, NSUI014, NSUI081
NSUI002_00312	164124..164273	284193..284342	NSUI010, NSUI014, NSUI081
NSUI002_00313	164274..164459	284358..284543	NSUI010, NSUI014, NSUI081
NSUI002_00314	164460..165743	284819..286102	NSUI010, NSUI014, NSUI018, NSUI036, NSUI081
NSUI002_00315	165744..166769	286183..287208	NSUI010, NSUI014, NSUI018, NSUI036, NSUI081
NSUI002_00317	166770..168254	290727..292211	NSUI010, NSUI014, NSUI018, NSUI036, NSUI081

NSUI002 Gene Locus ID	Gene Position in Core Genome	Gene Position NSUI002 Genome	Strains Showing Recombination
NSUI002_00321	168255..168449	295228..295422	NSUI010, NSUI014, NSUI018, NSUI036, NSUI081
NSUI002_00322	168450..168728	295444..295722	NSUI010, NSUI014, NSUI018, NSUI036, NSUI081
NSUI002_00323	168729..170390	295715..297376	NSUI010, NSUI014, NSUI018, NSUI036, NSUI081
NSUI002_00325	170391..171668	298096..299373	NSUI010, NSUI014, NSUI018, NSUI036, NSUI081
NSUI002_00340	186939..188386	316688..318136	NSUI058, NSUI079
NSUI002_00341	188387..189262	318261..319136	NSUI058, NSUI079
NSUI002_00342	189263..189934	319227..319898	NSUI058, NSUI079
NSUI002_00344	189935..190441	320417..320923	NSUI058, NSUI079
NSUI002_00345	190442..190657	320925..321140	NSUI058, NSUI079
NSUI002_00346	190658..191182	321142..321666	NSUI058, NSUI079
NSUI002_00347	191183..193051	321801..323669	NSUI058, NSUI079
NSUI002_00348	193052..193507	323831..324286	NSUI058, NSUI079
NSUI002_00349	193508..194257	324453..325202	NSUI058, NSUI079
NSUI002_00350	194258..195019	325192..325953	NSUI058, NSUI079
NSUI002_00352	195020..195586	326409..326975	NSUI058, NSUI079
NSUI002_00353	195587..196201	326975..327589	NSUI058, NSUI079
NSUI002_00359	196202..196711	332578..333087	NSUI058, NSUI079
NSUI002_00360	196712..197830	333230..334348	NSUI058, NSUI079
NSUI002_00361	197831..198850	334358..335377	NSUI058, NSUI079
NSUI002_00362	198851..200134	335599..336882	NSUI058, NSUI079
NSUI002_00364	200135..201055	337927..338847	NSUI058, NSUI079
NSUI002_00365	201056..204211	338881..342036	NSUI058, NSUI079
NSUI002_00490	261884..264352	456951..459419	NSUI095
NSUI002_00491	264353..265429	459581..460657	NSUI095

NSUI002 Gene Locus ID	Gene Position in Core Genome	Gene Position NSUI002 Genome	Strains Showing Recombination
NSUI002_00492	265430..266668	460722..461960	NSUI095
NSUI002_00493	266669..267454	461970..462755	NSUI095
NSUI002_00494	267455..267859	462778..463182	NSUI095
NSUI002_00495	267860..268711	463309..464160	NSUI095
NSUI002_00496	268712..269971	464259..465518	NSUI095
NSUI002_00517	284042..284548	488011..488517	NSUI031
NSUI002_00518	284549..284956	488477..488884	NSUI031
NSUI002_00520	284957..285244	489286..489573	NSUI031
NSUI002_00521	285245..285451	489690..489896	NSUI031
NSUI002_00526	285452..285979	493471..493998	NSUI031
NSUI002_00531	285980..286471	497763..498254	NSUI031
NSUI002_00532	286472..287299	498245..499072	NSUI031
NSUI002_00533	287300..288622	499084..500409	NSUI031
NSUI002_00534	288623..289981	500476..501834	NSUI031
NSUI002_00534	288623..289981	500476..501834	NSUI018, NSUI020, NSUI022, NSUI023, NSUI024, NSUI025, NSUI026, NSUI029, NSUI032, NSUI036
NSUI002_00535	289982..290164	501900..502082	NSUI018, NSUI020, NSUI022, NSUI023, NSUI024, NSUI025, NSUI026, NSUI029, NSUI032, NSUI036
NSUI002_00535	289982..290164	501900..502082	NSUI025
NSUI002_00537	290165..290710	503605..504150	NSUI025
NSUI002_00538	290711..291160	504213..504662	NSUI025
NSUI002_00539	291161..291658	504965..505462	NSUI025
NSUI002_00540	291659..292840	505459..506640	NSUI025
NSUI002_00541	292841..294187	506655..508001	NSUI025
NSUI002_00544	294188..295507	509165..510484	NSUI025

NSUI002 Gene Locus ID	Gene Position in Core Genome	Gene Position NSUI002 Genome	Strains Showing Recombination
NSUI002_00545	295508..295885	510668..511045	NSUI025
NSUI002_00546	295886..296773	511067..511954	NSUI025
NSUI002_00547	296774..297748	511951..512925	NSUI025
NSUI002_00548	297749..298666	512922..513839	NSUI025
NSUI002_00549	298667..299362	514123..514818	NSUI025
NSUI002_00550	299363..300592	515081..516310	NSUI025
NSUI002_00551	300593..301033	516310..516750	NSUI025
NSUI002_00552	301034..302047	516769..517782	NSUI025
NSUI002_00554	302048..303550	519190..520692	NSUI025
NSUI002_00555	303551..304891	520811..522151	NSUI025
NSUI002_00556	304892..309151	522522..526781	NSUI025
NSUI002_00557	309152..309625	527074..527547	NSUI025
NSUI002_00558	309626..310654	527596..528624	NSUI025
NSUI002_00559	310655..311359	528972..529676	NSUI025
NSUI002_00559	310655..311359	528972..529676	NSUI025, NSUI095
NSUI002_00563	311360..311704	532088..532432	NSUI025, NSUI095
NSUI002_00564	311705..311830	532904..533029	NSUI025, NSUI095
NSUI002_00565	311831..313366	533046..534581	NSUI025, NSUI095
NSUI002_00566	313367..314608	534578..535819	NSUI025, NSUI095
NSUI002_00567	314609..314848	535862..536101	NSUI025, NSUI095
NSUI002_00568	314849..316114	536094..537359	NSUI025, NSUI095
NSUI002_00570	316115..317071	538616..539572	NSUI025, NSUI095
NSUI002_00571	317072..317788	539711..540427	NSUI025, NSUI095
NSUI002_00572	317789..318268	540441..540920	NSUI025, NSUI095
NSUI002_00573	318269..319270	540939..541940	NSUI025, NSUI095
NSUI002_00574	319271..320296	541937..542962	NSUI025, NSUI095
NSUI002_00575	320297..321226	542976..543905	NSUI025, NSUI095

NSUI002 Gene Locus ID	Gene Position in Core Genome	Gene Position NSUI002 Genome	Strains Showing Recombination
NSUI002_00576	321227..322027	543902..544702	NSUI025, NSUI095
NSUI002_00577	322028..323473	544855..546300	NSUI025, NSUI095
NSUI002_00578	323474..324220	546446..547192	NSUI025, NSUI095
NSUI002_00582	324221..324877	550467..551123	NSUI025, NSUI095
NSUI002_00583	324878..325705	551217..552044	NSUI025, NSUI095
NSUI002_00584	325706..326977	552050..553321	NSUI025, NSUI095
NSUI002_00585	326978..327844	553358..554224	NSUI025, NSUI095
NSUI002_00586	327845..328483	554399..555037	NSUI025, NSUI095
NSUI002_00587	328484..329368	555034..555918	NSUI025, NSUI095
NSUI002_00588	329369..329686	555938..556255	NSUI025, NSUI095
NSUI002_00589	329687..330550	556257..557120	NSUI025, NSUI095
NSUI002_00590	330551..331642	557590..558681	NSUI025, NSUI095
NSUI002_00591	331643..332200	558681..559238	NSUI025, NSUI095
NSUI002_00594	332201..332554	561286..561639	NSUI025, NSUI095
NSUI002_00595	332555..333454	561656..562555	NSUI025, NSUI095
NSUI002_00596	333455..334282	562855..563682	NSUI025, NSUI095
NSUI002_00613	350003..350704	581571..582272	NSUI095
NSUI002_00614	350705..352375	582770..584440	NSUI095
NSUI002_00615	352376..353185	584484..585293	NSUI095
NSUI002_00618	353186..354027	587068..587919	NSUI095
NSUI002_00619	354028..354645	588077..588694	NSUI095
NSUI002_00620	354646..355155	588695..589204	NSUI095
NSUI002_00621	355156..355809	589194..589847	NSUI095
NSUI002_00622	355810..356097	589858..590145	NSUI095
NSUI002_00633	356098..357174	600894..601970	NSUI095
NSUI002_00634	357175..358110	602043..602978	NSUI095
NSUI002_00637	358111..358743	605249..605881	NSUI095

NSUI002 Gene Locus ID	Gene Position in Core Genome	Gene Position NSUI002 Genome	Strains Showing Recombination
NSUI002_00638	358744..360036	605938..607230	NSUI095
NSUI002_00671	375577..378450	633715..636588	NSUI095
NSUI002_00673	378451..379500	637079..638128	NSUI095
NSUI002_00674	379501..380493	638213..639205	NSUI095
NSUI002_00675	380494..382341	639338..641185	NSUI095
NSUI002_00676	382342..382590	641203..641451	NSUI095
NSUI002_00677	382591..382851	642158..642418	NSUI095, NSUI101
NSUI002_00678	382852..383410	642411..643001	NSUI095, NSUI101
NSUI002_00681	383411..384847	648118..649554	NSUI095
NSUI002_00682	384848..385717	649668..650537	NSUI095
NSUI002_00721	411608..412855	686479..687726	NSUI025
NSUI002_00722	412856..413920	687852..688916	NSUI025
NSUI002_00724	413921..415411	691625..693115	NSUI025
NSUI002_00725	415412..416038	693218..693844	NSUI025
NSUI002_00726	416039..416521	693847..694329	NSUI025
NSUI002_00728	416522..417070	695274..695822	NSUI025
NSUI002_00730	417071..417574	696967..697470	NSUI025
NSUI002_00731	417575..417940	697467..697832	NSUI025
NSUI002_00732	417941..419155	697969..699183	NSUI025
NSUI002_00733	419156..421852	699204..701900	NSUI025
NSUI002_00734	421853..422290	702081..702518	NSUI025
NSUI002_00735	422291..423256	702505..703470	NSUI025
NSUI002_00750	435191..437335	718369..720513	NSUI010, NSUI018, NSUI081
NSUI002_00751	437336..437806	720510..720980	NSUI010, NSUI018, NSUI081
NSUI002_00753	437807..437902	721331..721426	NSUI010, NSUI018, NSUI081
NSUI002_00758	437903..438604	727115..727816	NSUI010, NSUI018, NSUI081
NSUI002_00761	438605..439102	730368..730865	NSUI010, NSUI018, NSUI081

NSUI002 Gene Locus ID	Gene Position in Core Genome	Gene Position NSUI002 Genome	Strains Showing Recombination
NSUI002_00762	439103..439504	730846..731247	NSUI010, NSUI018, NSUI081
NSUI002_00763	439505..440404	731264..732163	NSUI010, NSUI036, NSUI081
NSUI002_00764	440405..441298	732785..733678	NSUI010, NSUI036, NSUI081
NSUI002_00765	441299..441901	733671..734273	NSUI010, NSUI036, NSUI081
NSUI002_00767	441902..442135	735661..735894	NSUI010, NSUI036, NSUI081
NSUI002_00768	442136..444505	736325..738694	NSUI010, NSUI018, NSUI036, NSUI081
NSUI002_00769	444506..444973	738697..739164	NSUI010, NSUI018, NSUI036, NSUI081, NSUI091
NSUI002_00770	444974..446023	739251..740300	NSUI010, NSUI018, NSUI036, NSUI081
NSUI002_00771	446024..446884	740448..741308	NSUI010, NSUI018, NSUI036, NSUI081, NSUI091
NSUI002_00775	446885..447220	746636..746971	NSUI010, NSUI018, NSUI036, NSUI081, NSUI091
NSUI002_00776	447221..447922	747060..747761	NSUI010, NSUI018, NSUI036, NSUI081, NSUI091
NSUI002_00777	447923..451261	747800..751138	NSUI003, NSUI007, NSUI009, NSUI010, NSUI011, NSUI014, NSUI015, NSUI016, NSUI017, NSUI018, NSUI019, NSUI020, NSUI021, NSUI022, NSUI023, NSUI024, NSUI026, NSUI027, NSUI028, NSUI029, NSUI030, NSUI031, NSUI032, NSUI036, NSUI067, NSUI081, NSUI091
NSUI002_00779	451262..451513	753023..753274	NSUI003, NSUI007, NSUI009, NSUI010, NSUI011, NSUI014, NSUI015, NSUI016, NSUI017, NSUI018, NSUI019, NSUI020, NSUI021, NSUI022, NSUI023, NSUI024, NSUI026, NSUI027, NSUI028, NSUI029, NSUI030, NSUI031, NSUI036, NSUI067,

NSUI002 Gene Locus ID	Gene Position in Core Genome	Gene Position NSUI002 Genome	Strains Showing Recombination
			NSUI081, NSUI091
NSUI002_00781	451514..451780	753552..753818	NSUI003, NSUI007, NSUI009, NSUI010, NSUI011, NSUI014, NSUI015, NSUI016, NSUI017, NSUI018, NSUI019, NSUI020, NSUI021, NSUI022, NSUI023, NSUI024, NSUI026, NSUI027, NSUI028, NSUI029, NSUI030, NSUI031, NSUI032, NSUI036, NSUI067, NSUI081, NSUI091
NSUI002_00782	451781..452872	754021..755112	NSUI003, NSUI007, NSUI009, NSUI010, NSUI011, NSUI014, NSUI015, NSUI016, NSUI017, NSUI018, NSUI019, NSUI020, NSUI021, NSUI022, NSUI023, NSUI024, NSUI025, NSUI026, NSUI027, NSUI028, NSUI029, NSUI030, NSUI031, NSUI032, NSUI036, NSUI067, NSUI081, NSUI091
NSUI002_00783	452873..454033	755116..756276	NSUI003, NSUI005, NSUI007, NSUI009, NSUI010, NSUI011, NSUI014, NSUI015, NSUI016, NSUI017, NSUI018, NSUI019, NSUI020, NSUI021, NSUI022, NSUI023, NSUI024, NSUI026, NSUI027, NSUI028, NSUI029, NSUI030, NSUI031, NSUI032, NSUI036, NSUI067, NSUI080, NSUI081, NSUI091
NSUI002_00784	454034..454873	756308..757147	NSUI003, NSUI005, NSUI007, NSUI009, NSUI011, NSUI014, NSUI015, NSUI016, NSUI017, NSUI018, NSUI019, NSUI020, NSUI021, NSUI022, NSUI023, NSUI024, NSUI026, NSUI027, NSUI028, NSUI029, NSUI030, NSUI031, NSUI032, NSUI036, NSUI067, NSUI080, NSUI081,

NSUI002 Gene Locus ID	Gene Position in Core Genome	Gene Position NSUI002 Genome	Strains Showing Recombination
			NSUI091
NSUI002_00786	454874..456109	759441..760676	NSUI005, NSUI014, NSUI018, NSUI020, NSUI022, NSUI023, NSUI024, NSUI025, NSUI031, NSUI032, NSUI036, NSUI080, NSUI081
NSUI002_00787	456110..456982	760813..761685	NSUI005, NSUI020, NSUI025, NSUI031, NSUI032, NSUI080
NSUI002_00788	456983..457813	761696..762526	NSUI005, NSUI025, NSUI032, NSUI080
NSUI002_00789	457814..459262	762595..764043	NSUI003, NSUI020, NSUI025, NSUI031, NSUI032, NSUI091
NSUI002_00790	459263..460879	764116..765732	NSUI003, NSUI020, NSUI025, NSUI031, NSUI091
NSUI002_00791	460880..461509	765815..766444	NSUI003, NSUI020, NSUI031, NSUI091
NSUI002_00792	461510..462100	766561..767151	NSUI020, NSUI031
NSUI002_00794	462101..463270	767681..768850	NSUI020, NSUI031
NSUI002_00795	463271..464158	769056..769943	NSUI020, NSUI031
NSUI002_00796	464159..465106	769946..770893	NSUI020, NSUI031
NSUI002_00796	464159..465106	769946..770893	NSUI025
NSUI002_00797	465107..465871	770893..771657	NSUI025
NSUI002_00798	465872..466582	771657..772367	NSUI025
NSUI002_00870	466583..467785	815815..817017	NSUI025
NSUI002_00934	515381..516484	886551..887654	NSUI095
NSUI002_00935	516485..516823	887664..888002	NSUI095
NSUI002_00960	516824..518614	908733..910523	NSUI095
NSUI002_00961	518615..518953	910690..911028	NSUI095
NSUI002_00962	518954..520321	911213..912580	NSUI095
NSUI002_00966	522602..523366	917372..918136	NSUI020, NSUI031

NSUI002 Gene Locus ID	Gene Position in Core Genome	Gene Position NSUI002 Genome	Strains Showing Recombination
NSUI002_00968	523367..524122	918981..919736	NSUI020, NSUI031
NSUI002_00972	524123..524866	921911..922654	NSUI020, NSUI031
NSUI002_00973	524867..526735	922810..924678	NSUI020, NSUI031
NSUI002_00974	526736..526864	924735..924863	NSUI020, NSUI031
NSUI002_00976	526865..527029	926754..926918	NSUI020, NSUI031
NSUI002_00978	527030..530296	931380..934646	NSUI020, NSUI031
NSUI002_00979	530297..533950	934646..938299	NSUI020, NSUI031
NSUI002_01047	582437..583396	1007521..1008480	NSUI018
NSUI002_01049	583397..583699	1008897..1009199	NSUI018
NSUI002_01050	583700..584539	1009252..1010091	NSUI018
NSUI002_01051	584540..585052	1010429..1010941	NSUI018
NSUI002_01052	585053..585226	1010944..1011117	NSUI018
NSUI002_01082	602912..603982	1037838..1038908	NSUI003, NSUI091
NSUI002_01084	603983..604922	1040562..1041503	NSUI003, NSUI091
NSUI002_01086	604923..605180	1042204..1042461	NSUI003, NSUI091
NSUI002_01087	605181..605420	1042451..1042690	NSUI003, NSUI091
NSUI002_01089	605421..606173	1044753..1045505	NSUI003, NSUI091
NSUI002_01096	609345..610172	1049142..1049969	NSUI091
NSUI002_01097	610173..611177	1050083..1051087	NSUI091
NSUI002_01098	611178..612014	1051152..1051988	NSUI091
NSUI002_01099	612015..613349	1052065..1053399	NSUI002, NSUI003, NSUI004, NSUI005, NSUI007, NSUI008, NSUI009, NSUI010, NSUI011, NSUI014, NSUI015, NSUI016, NSUI017, NSUI018, NSUI019, NSUI020, NSUI021, NSUI022, NSUI023, NSUI024, NSUI025, NSUI026, NSUI031, NSUI036, NSUI058, NSUI062, NSUI064, NSUI073, NSUI074, NSUI076, NSUI079, NSUI080, NSUI081,

NSUI002 Gene Locus ID	Gene Position in Core Genome	Gene Position NSUI002 Genome	Strains Showing Recombination
			NSUI083, NSUI084, NSUI087, NSUI090, NSUI091, NSUI098, NSUI101
NSUI002_01100	613350..614099	1053595..1054344	NSUI002, NSUI003, NSUI004, NSUI005, NSUI007, NSUI008, NSUI009, NSUI010, NSUI011, NSUI014, NSUI015, NSUI016, NSUI017, NSUI018, NSUI019, NSUI020, NSUI021, NSUI022, NSUI023, NSUI024, NSUI025, NSUI026, NSUI031, NSUI036, NSUI058, NSUI062, NSUI064, NSUI073, NSUI074, NSUI076, NSUI079, NSUI080, NSUI081, NSUI083, NSUI084, NSUI087, NSUI090, NSUI091, NSUI098, NSUI101
NSUI002_01159	614100..614460	1119925..1120290	NSUI002, NSUI003, NSUI004, NSUI005, NSUI007, NSUI008, NSUI009, NSUI010, NSUI011, NSUI014, NSUI015, NSUI016, NSUI017, NSUI018, NSUI019, NSUI020, NSUI021, NSUI022, NSUI023, NSUI024, NSUI025, NSUI026, NSUI031, NSUI036, NSUI058, NSUI062, NSUI064, NSUI073, NSUI074, NSUI076, NSUI079, NSUI080, NSUI081, NSUI083, NSUI084, NSUI087, NSUI090, NSUI091, NSUI098, NSUI101
NSUI002_01170	614461..614680	1128643..1128870	NSUI002, NSUI004, NSUI005, NSUI007, NSUI008, NSUI009, NSUI010, NSUI011, NSUI015, NSUI016, NSUI017, NSUI018, NSUI019, NSUI021, NSUI022, NSUI023, NSUI024, NSUI025, NSUI058, NSUI062, NSUI064, NSUI073, NSUI074, NSUI076, NSUI079, NSUI080, NSUI081,

NSUI002 Gene Locus ID	Gene Position in Core Genome	Gene Position NSUI002 Genome	Strains Showing Recombination
			NSUI083, NSUI084, NSUI087, NSUI090, NSUI098, NSUI101
NSUI002_01193	614681..615232	1154029..1154580	NSUI004, NSUI005, NSUI007, NSUI008, NSUI009, NSUI010, NSUI011, NSUI014, NSUI015, NSUI016, NSUI017, NSUI019, NSUI021, NSUI058, NSUI064, NSUI073, NSUI074, NSUI079, NSUI080, NSUI081, NSUI084, NSUI090, NSUI098, NSUI101
NSUI002_01194	615233..615416	1154564..1154755	NSUI004, NSUI005, NSUI007, NSUI008, NSUI009, NSUI010, NSUI011, NSUI014, NSUI015, NSUI016, NSUI017, NSUI019, NSUI021, NSUI058, NSUI064, NSUI073, NSUI074, NSUI079, NSUI080, NSUI081, NSUI084, NSUI090, NSUI098, NSUI101
NSUI002_01214	615417..615782	1177107..1177472	NSUI004, NSUI005, NSUI007, NSUI008, NSUI009, NSUI010, NSUI011, NSUI014, NSUI015, NSUI016, NSUI017, NSUI019, NSUI021, NSUI058, NSUI064, NSUI073, NSUI074, NSUI079, NSUI080, NSUI081, NSUI084, NSUI090, NSUI098, NSUI101
NSUI002_01215	615783..616277	1177538..1178032	NSUI010, NSUI011, NSUI014, NSUI019, NSUI021, NSUI064, NSUI073, NSUI081, NSUI090, NSUI098
NSUI002_01225	623673..624452	1186954..1187733	NSUI036
NSUI002_01226	624453..625304	1187720..1188571	NSUI036
NSUI002_01286	654855..655832	1253419..1254396	NSUI014
NSUI002_01287	655833..656948	1254407..1255522	NSUI014
NSUI002_01288	656949..657293	1255524..1255868	NSUI014
NSUI002_01289	657294..657497	1255870..1256073	NSUI014

NSUI002 Gene Locus ID	Gene Position in Core Genome	Gene Position NSUI002 Genome	Strains Showing Recombination
NSUI002_01290	657498..658136	1256208..1256846	NSUI014
NSUI002_01292	658137..658817	1257539..1258219	NSUI014
NSUI002_01293	658818..659567	1258333..1259082	NSUI014
NSUI002_01337	688548..689462	1298388..1299302	NSUI018
NSUI002_01338	689463..689780	1299433..1299750	NSUI018
NSUI002_01340	689781..690355	1300871..1301449	NSUI018
NSUI002_01341	690356..690817	1301436..1301897	NSUI018
NSUI002_01343	690818..691177	1302325..1302684	NSUI018
NSUI002_01344	691178..691603	1302704..1303129	NSUI018
NSUI002_01347	691604..692842	1304561..1305799	NSUI018
NSUI002_01443	766739..767776	1407854..1408891	NSUI022, NSUI023, NSUI024
NSUI002_01444	767777..768802	1408901..1409926	NSUI022, NSUI023, NSUI024
NSUI002_01445	768803..769672	1409972..1410841	NSUI022, NSUI023, NSUI024
NSUI002_01446	769673..770740	1410854..1411921	NSUI022, NSUI023, NSUI024
NSUI002_01447	770741..771541	1412125..1412925	NSUI022, NSUI023, NSUI024
NSUI002_01522	816152..817813	1493323..1494984	NSUI025
NSUI002_01523	817814..818218	1495240..1495644	NSUI025
NSUI002_01524	818219..818704	1495650..1496135	NSUI025
NSUI002_01525	818705..819463	1496318..1497076	NSUI025
NSUI002_01526	819464..820330	1497073..1497939	NSUI025
NSUI002_01527	820331..821332	1497962..1498963	NSUI025
NSUI002_01529	821333..823174	1501159..1503000	NSUI025
NSUI002_01532	823175..823891	1505840..1506556	NSUI025
NSUI002_01533	823892..825121	1506560..1507789	NSUI025
NSUI002_01534	825122..826288	1507779..1508945	NSUI025
NSUI002_01538	828122..828382	1511175..1511435	NSUI010, NSUI014, NSUI018, NSUI036, NSUI081

NSUI002 Gene Locus ID	Gene Position in Core Genome	Gene Position NSUI002 Genome	Strains Showing Recombination
NSUI002_01539	828383..828604	1511437..1511658	NSUI010, NSUI014, NSUI018, NSUI036, NSUI081
NSUI002_01558	828605..829057	1522881..1523333	NSUI010, NSUI014, NSUI018, NSUI036, NSUI081
NSUI002_01561	829058..829708	1524796..1525446	NSUI010, NSUI014, NSUI018, NSUI036, NSUI081
NSUI002_01562	829709..830344	1525477..1526112	NSUI010, NSUI014, NSUI018, NSUI036, NSUI081
NSUI002_01563	830345..832069	1526164..1527888	NSUI010, NSUI014, NSUI018, NSUI036, NSUI081
NSUI002_01564	832070..834022	1527977..1529929	NSUI010, NSUI014, NSUI018, NSUI036, NSUI081
NSUI002_01565	834023..834562	1530111..1530650	NSUI010, NSUI014, NSUI018, NSUI036, NSUI081
NSUI002_01566	834563..835780	1530664..1531881	NSUI010, NSUI014, NSUI018, NSUI036, NSUI081
NSUI002_01568	835781..835969	1533896..1534084	NSUI010, NSUI014, NSUI018, NSUI036, NSUI081
NSUI002_01569	835970..836425	1534117..1534572	NSUI010, NSUI014, NSUI018, NSUI036, NSUI081
NSUI002_01570	836426..837346	1534582..1535502	NSUI010, NSUI018, NSUI081
NSUI002_01571	837347..838201	1535512..1536366	NSUI010, NSUI018, NSUI081
NSUI002_01586	849731..850720	1551258..1552247	NSUI010, NSUI018, NSUI081
NSUI002_01587	850721..851266	1552404..1552949	NSUI010, NSUI018, NSUI081
NSUI002_01588	851267..851644	1552950..1553327	NSUI010, NSUI018, NSUI081
NSUI002_01589	851645..851998	1553412..1553765	NSUI010, NSUI018, NSUI081
NSUI002_01590	851999..852802	1553908..1554711	NSUI010, NSUI018, NSUI081
NSUI002_01592	852803..854347	1556446..1557990	NSUI010, NSUI014, NSUI018, NSUI036, NSUI081
NSUI002_01593	854348..856684	1558227..1560563	NSUI010, NSUI014, NSUI018, NSUI036, NSUI081

NSUI002 Gene Locus ID	Gene Position in Core Genome	Gene Position NSUI002 Genome	Strains Showing Recombination
NSUI002_01614	869924..870109	1579980..1580165	NSUI032
NSUI002_01615	870110..870484	1580615..1580989	NSUI032
NSUI002_01616	870485..871798	1581004..1582317	NSUI032
NSUI002_01626	880706..882076	1595381..1596751	NSUI005, NSUI032, NSUI080
NSUI002_01630	882077..882688	1599579..1600190	NSUI005, NSUI032, NSUI080
NSUI002_01631	882689..883096	1600313..1600720	NSUI005, NSUI032, NSUI080
NSUI002_01632	883097..883696	1600832..1601434	NSUI005, NSUI032, NSUI080
NSUI002_01633	883697..883975	1601421..1601699	NSUI005, NSUI032, NSUI080
NSUI002_01635	883976..884569	1603858..1604451	NSUI005, NSUI032, NSUI080
NSUI002_01636	884570..884953	1604529..1604912	NSUI005, NSUI032, NSUI080
NSUI002_01637	884954..887530	1605000..1607576	NSUI005, NSUI032, NSUI080
NSUI002_01638	887531..888283	1607961..1608713	NSUI005, NSUI032, NSUI080
NSUI002_01639	888284..888814	1609232..1609762	NSUI005, NSUI032, NSUI080
NSUI002_01640	888815..889663	1609759..1610607	NSUI005, NSUI032, NSUI080
NSUI002_01642	889664..890353	1612226..1612915	NSUI005, NSUI080
NSUI002_01643	890354..890674	1612924..1613244	NSUI005, NSUI080
NSUI002_01644	890675..891220	1613255..1613800	NSUI005, NSUI080
NSUI002_01645	891221..892603	1613809..1615191	NSUI005, NSUI080
NSUI002_01646	892604..893074	1615368..1615838	NSUI005, NSUI080
NSUI002_01647	893075..893626	1615835..1616386	NSUI005, NSUI080
NSUI002_01648	893627..894325	1616399..1617097	NSUI005, NSUI080
NSUI002_01649	894326..894562	1617090..1617326	NSUI005, NSUI080
NSUI002_01650	894563..895357	1617379..1618173	NSUI005, NSUI080
NSUI002_01657	900290..901459	1623552..1624721	NSUI058, NSUI079
NSUI002_01658	901460..902584	1624736..1625860	NSUI058, NSUI079
NSUI002_01659	902585..903346	1626008..1626769	NSUI058, NSUI079
NSUI002_01660	903347..904855	1626779..1628290	NSUI058, NSUI079

NSUI002 Gene Locus ID	Gene Position in Core Genome	Gene Position NSUI002 Genome	Strains Showing Recombination
NSUI002_01661	904856..905587	1628290..1629021	NSUI058, NSUI079
NSUI002_01662	905588..906034	1629014..1629460	NSUI058, NSUI079
NSUI002_01663	906035..906571	1629774..1630310	NSUI058, NSUI079
NSUI002_01664	906572..906985	1630294..1630707	NSUI058, NSUI079
NSUI002_01665	906986..907762	1630718..1631494	NSUI058, NSUI079
NSUI002_01666	907763..908695	1631496..1632428	NSUI058, NSUI079
NSUI002_01667	908696..909637	1632646..1633587	NSUI058, NSUI079
NSUI002_01669	909638..910399	1633997..1634758	NSUI058, NSUI079
NSUI002_01670	910400..910834	1634828..1635262	NSUI058, NSUI079
NSUI002_01671	910835..912976	1635249..1637390	NSUI058, NSUI079
NSUI002_01672	912977..913828	1637466..1638317	NSUI058, NSUI079
NSUI002_01686	922439..924304	1653899..1655764	NSUI025
NSUI002_01687	924305..925036	1655907..1656644	NSUI025
NSUI002_01688	925037..926071	1656646..1657680	NSUI025
NSUI002_01688	925037..926071	1656646..1657680	NSUI095
NSUI002_01689	926072..927028	1657745..1658701	NSUI095
NSUI002_01690	927029..927217	1658667..1658855	NSUI095
NSUI002_01692	927218..927715	1660717..1661214	NSUI025
NSUI002_01693	927716..927985	1661279..1661548	NSUI025
NSUI002_01696	927986..928615	1662176..1662805	NSUI025
NSUI002_01697	928616..929602	1662888..1663874	NSUI025
NSUI002_01698	929603..930685	1663864..1664946	NSUI025
NSUI002_01699	930686..932083	1665013..1666410	NSUI025
NSUI002_01701	932084..932776	1667189..1667881	NSUI025
NSUI002_01703	932777..933931	1669320..1670474	NSUI025
NSUI002_01749	965834..966268	1715888..1716322	NSUI020, NSUI031
NSUI002_01750	966269..966691	1716454..1716876	NSUI020, NSUI031

NSUI002 Gene Locus ID	Gene Position in Core Genome	Gene Position NSUI002 Genome	Strains Showing Recombination
NSUI002_01751	966692..968491	1716866..1718665	NSUI020, NSUI031
NSUI002_01752	968492..969145	1718889..1719542	NSUI020, NSUI031
NSUI002_01758	971540..972271	1723793..1724524	NSUI003, NSUI091
NSUI002_01759	972272..972730	1724521..1724979	NSUI003, NSUI091
NSUI002_01760	972731..973258	1724976..1725503	NSUI003, NSUI091
NSUI002_01761	973259..974269	1725476..1726486	NSUI003, NSUI091
NSUI002_01762	974270..975064	1726483..1727277	NSUI003, NSUI091
NSUI002_01865	1042922..1043806	1831447..1832331	NSUI095
NSUI002_01868	1043807..1044772	1836037..1837002	NSUI095
NSUI002_01869	1044773..1045192	1837081..1837500	NSUI095
NSUI002_01870	1045193..1045585	1837490..1837882	NSUI095
NSUI002_01871	1045586..1046143	1837910..1838467	NSUI095
NSUI002_01872	1046144..1047205	1838531..1839592	NSUI095
NSUI002_01873	1047206..1050031	1839691..1842516	NSUI095
NSUI002_01874	1050032..1050976	1843355..1844299	NSUI095
NSUI002_01875	1050977..1051633	1844315..1844971	NSUI095
NSUI002_01876	1051634..1051873	1845362..1845601	NSUI095
NSUI002_01878	1051874..1052164	1846140..1846430	NSUI095
NSUI002_01879	1052165..1053154	1846602..1847591	NSUI095
NSUI002_01881	1053155..1053772	1849111..1849728	NSUI095
NSUI002_01930	1085420..1086661	1898901..1900142	NSUI011
NSUI002_01931	1086662..1088077	1900416..1901831	NSUI011
NSUI002_01932	1088078..1088518	1902181..1902621	NSUI011
NSUI002_01934	1088519..1089781	1903836..1905098	NSUI011
NSUI002_01955	1101425..1102891	1925249..1926715	NSUI031
NSUI002_01956	1102892..1104325	1927047..1928480	NSUI031
NSUI002_01981	1107416..1107808	1950920..1951312	NSUI002, NSUI064, NSUI073,

NSUI002 Gene Locus ID	Gene Position in Core Genome	Gene Position NSUI002 Genome	Strains Showing Recombination
			NSUI083, NSUI087, NSUI090, NSUI098
NSUI002_01982	1107809..1108255	1951332..1951778	NSUI002, NSUI064, NSUI073, NSUI083, NSUI087, NSUI090, NSUI098
NSUI002_01984	1108256..1108777	1953102..1953623	NSUI002, NSUI064, NSUI073, NSUI083, NSUI087, NSUI090, NSUI098
NSUI002_02010	1122167..1122952	1975642..1976427	NSUI095
NSUI002_02011	1122953..1123546	1976490..1977083	NSUI095
NSUI002_02013	1123547..1124599	1978471..1979523	NSUI095
NSUI002_02015	1124600..1125853	1981553..1982806	NSUI095
NSUI002_02016	1125854..1126777	1982838..1983761	NSUI095
NSUI002_02023	1130201..1130872	1988361..1989032	NSUI095
NSUI002_02024	1130873..1135264	1989044..1993435	NSUI095
NSUI002_02025	1135265..1136254	1993559..1994548	NSUI095
NSUI002_02026	1136255..1136800	1994559..1995104	NSUI095
NSUI002_02027	1136801..1137790	1995252..1996241	NSUI095
NSUI002_02028	1137791..1138366	1996252..1996827	NSUI095
NSUI002_02029	1138367..1138741	1996824..1997198	NSUI095
NSUI002_02030	1138742..1139452	1997208..1997918	NSUI095
NSUI002_02040	1151972..1152976	2012292..2013296	NSUI020, NSUI031
NSUI002_02041	1152977..1153468	2013397..2013888	NSUI020, NSUI031
NSUI002_02042	1153469..1155922	2014006..2016459	NSUI020, NSUI031
NSUI002_02043	1155923..1156375	2016463..2016915	NSUI020, NSUI031
NSUI002_02047	1156376..1157416	2021360..2022400	NSUI020, NSUI031
NSUI002_02048	1157417..1158193	2022653..2023429	NSUI020, NSUI031
NSUI002_02052	1158194..1158733	2030164..2030703	NSUI003, NSUI020, NSUI031, NSUI091

NSUI002 Gene Locus ID	Gene Position in Core Genome	Gene Position NSUI002 Genome	Strains Showing Recombination
NSUI002_02053	1158734..1158910	2030813..2030989	NSUI003, NSUI020, NSUI031, NSUI091
NSUI002_02054	1158911..1159063	2030999..2031151	NSUI003, NSUI020, NSUI031, NSUI091
NSUI002_02056	1159064..1160032	2033551..2034519	NSUI003, NSUI020, NSUI031, NSUI091
NSUI002_02085	1160033..1160845	2062055..2062867	NSUI003, NSUI020, NSUI031, NSUI091
NSUI002_02087	1160846..1161787	2063828..2064769	NSUI003, NSUI020, NSUI031, NSUI091
NSUI002_02089	1161788..1162420	2065998..2066630	NSUI003, NSUI020, NSUI031, NSUI091
NSUI002_02090	1162421..1163080	2066623..2067282	NSUI003, NSUI020, NSUI031, NSUI091
NSUI002_02091	1163081..1163956	2067297..2068172	NSUI003, NSUI091
NSUI002_02095	1163957..1165676	2072976..2074706	NSUI003, NSUI091
NSUI002_02096	1165677..1166549	2075069..2075941	NSUI003, NSUI091
NSUI002_02097	1166550..1167080	2075961..2076491	NSUI003, NSUI091
NSUI002_02098	1167081..1167338	2076505..2076762	NSUI003, NSUI091
NSUI002_02099	1167339..1167599	2076764..2077024	NSUI003, NSUI091
NSUI002_02101	1167600..1167968	2077559..2077927	NSUI003, NSUI091
NSUI002_02102	1167969..1168559	2077920..2078510	NSUI003, NSUI091
NSUI002_02103	1168560..1169342	2078473..2079255	NSUI003, NSUI091
NSUI002_02104	1169343..1169480	2079363..2079500	NSUI003, NSUI091
NSUI002_02105	1169481..1170473	2079668..2080663	NSUI003, NSUI020, NSUI031, NSUI091
NSUI002_02106	1170474..1171286	2080683..2081495	NSUI003, NSUI020, NSUI031, NSUI091
NSUI002_02107	1171287..1171646	2081479..2081838	NSUI003, NSUI020, NSUI031, NSUI091

NSUI002 Gene Locus ID	Gene Position in Core Genome	Gene Position NSUI002 Genome	Strains Showing Recombination
NSUI002_02108	1171647..1173031	2081909..2083303	NSUI003, NSUI091
NSUI002_02109	1173032..1174225	2083317..2084510	NSUI003, NSUI091
NSUI002_02109	1173032..1174225	2083317..2084510	NSUI003, NSUI020, NSUI031, NSUI091
NSUI002_02110	1174226..1174774	2084703..2085251	NSUI003, NSUI020, NSUI031, NSUI091
NSUI002_02115	1174775..1176055	2089032..2090312	NSUI003, NSUI020, NSUI031, NSUI091
NSUI002_02205	1229807..1230658	2180922..2181773	NSUI003, NSUI091
NSUI002_02207	1230659..1230832	2182606..2182779	NSUI003, NSUI091
NSUI002_02210	1230833..1232521	2186134..2187822	NSUI003, NSUI091
NSUI002_02212	1232522..1233046	2188970..2189494	NSUI003, NSUI091
NSUI002_02213	1233047..1233228	2189606..2189788	NSUI003, NSUI091
NSUI002_02214	1233229..1234746	2189829..2191346	NSUI003, NSUI091
NSUI002_02215	1234747..1236006	2191650..2192909	NSUI003, NSUI091
NSUI002_02216	1236007..1237296	2193128..2194417	NSUI003, NSUI091
NSUI002_02219	1237297..1238280	2196113..2197096	NSUI003, NSUI091
