# Supplementary material for: Complex Population Structure and Virulence Differences among Serotype 2 Streptococcus suis Strains Belonging to Sequence Type 28
Source: PLoS One. 2015 Sep 16;10(9):e0137760. doi: 10.1371/journal.pone.0137760 (PMC4574206; doi:10.1371/journal.pone.0137760)
Supplement: S4 Table — (PDF) [file pone.0137760.s007.pdf]

S4 Table. Common ortholog gene clusters among clade I ST28 *Streptococcus suis* strains.

Cluster Number	Example Gene	Predicted Translated Product	Present also in			
			clade II	clade III	clade IV	clade V
55	NSUI002_00629	formate acetyltransferase	Yes	Yes	Yes	No
177	NSUI002_01073	GTPase subunit of restriction endonuclease	Yes	Yes	Yes	No
360	NSUI002_00628	phosphotransferase system cellobiose-specific component IIC	Yes	Yes	Yes	No
514	NSUI002_00723	hypothetical protein	Yes	Yes	Yes	No
525	NSUI002_01339	membrane protein	Yes	Yes	Yes	No
543	NSUI002_00632	glycerol dehydrogenase	Yes	Yes	Yes	No
580	NSUI002_01495	CRISPR-associated protein Cas7	Yes	Yes	Yes	No
667	NSUI002_00625	transcriptional regulator	Yes	Yes	Yes	No
689	NSUI002_00631	glycerol dehydrogenase	Yes	Yes	Yes	No
714	NSUI002_01497	CRISPR-associated protein Cas1	Yes	Yes	Yes	No
804	NSUI002_00527	putative ATPase (AAA+ superfamily)	Yes	Yes	Yes	No
911	NSUI002_00623	pyruvate-formate lyase-activating enzyme	Yes	Yes	Yes	No
1052	NSUI002_00624	sugar metabolism transcriptional regulator	Yes	Yes	Yes	No
1108	NSUI002_00324	cobalt ABC transporter ATPase	Yes	Yes	Yes	No
1155	NSUI002_00630	fructose-6-phosphate aldolase	Yes	Yes	Yes	No
1290	NSUI002_01306	16S RNA methylase RsmC	Yes	Yes	Yes	No
1300	NSUI002_01250	IgA-specific zinc metalloproteinase	Yes	Yes	Yes	No
1451	NSUI002_01705	histone acetyltransferase HPA2-like acetyltransferase	Yes	Yes	Yes	No
1476	NSUI002_02100	NTP pyrophosphohydrolase including oxidative damage repair enzymes	Yes	Yes	Yes	No
1493	NSUI002_00343	putative lipoprotein	Yes	Yes	Yes	No
1618	NSUI002_00249	membrane protein	Yes	Yes	Yes	No
1698	NSUI002_01496	CRISPR-associated protein Cas2	Yes	Yes	Yes	No
1729	NSUI002_00626	phosphotransferase system cellobiose-specific component IIA	Yes	Yes	Yes	No
1730	NSUI002_01424	hypothetical protein	Yes	Yes	Yes	No
1752	NSUI002_00627	phosphotransferase system	Yes	Yes	Yes	No

Cluster Number	Example Gene	Predicted Translated Product	Present also in			
			clade II	clade III	clade IV	clade V
		cellobiose-specific component IIB				
1780	NSUI002_01802	membrane protein	Yes	Yes	Yes	No
1824	NSUI002_00467	hypothetical protein	Yes	Yes	Yes	No
1928	NSUI002_00616	pyridine nucleotide-disulfide oxidoreductase	Yes	Yes	Yes	No
1947	NSUI002_00393	hypothetical protein	Yes	Yes	Yes	No
1968	NSUI002_00470	hypothetical protein	Yes	Yes	Yes	No
147	NSUI002_02045	LPXTG cell wall surface protein	Yes	Yes	No	Yes
216	NSUI002_01673	LPXTG-motif cell wall anchor domain-containing protein	Yes	Yes	No	Yes
335	NSUI002_00222	phosphotransferase system cellobiose-specific component IIC	Yes	Yes	No	Yes
599	NSUI002_01273	integrase family protein	Yes	Yes	No	Yes
647	NSUI002_01266	tagatose 1,6-diphosphate aldolase	Yes	Yes	No	Yes
750	NSUI002_01267	tagatose-6-phosphate kinase	Yes	Yes	No	Yes
836	NSUI002_01547	DNA replication protein, putative	Yes	Yes	No	Yes
865	NSUI002_01546	GTP-binding protein	Yes	Yes	No	Yes
951	NSUI002_01271	hypothetical protein	Yes	Yes	No	Yes
1015	NSUI002_01270	sugar metabolism transcriptional regulator	Yes	Yes	No	Yes
1305	NSUI002_01274	hypothetical protein	Yes	Yes	No	Yes
1312	NSUI002_01187	parvulin-like peptidyl-prolyl isomerase	Yes	Yes	No	Yes
1343	NSUI002_01555	transcriptional regulator	Yes	Yes	No	Yes
1345	NSUI002_01081	ATP-binding membrane protein	Yes	Yes	No	Yes
1358	NSUI002_02049	adenylate kinase	Yes	Yes	No	Yes
1366	NSUI002_01544	phage protein	Yes	Yes	No	Yes
1380	NSUI002_01268	galactose-6-phosphate isomerase subunit LacB	Yes	Yes	No	Yes
1401	NSUI002_02111	small molecule binding protein	Yes	Yes	No	Yes
1465	NSUI002_01541	phage-like protein	Yes	Yes	No	Yes
1542	NSUI002_01269	ribose 5-phosphate isomerase RpiB	Yes	Yes	No	Yes
1567	NSUI002_00255	hypothetical protein	Yes	Yes	No	Yes
1590	NSUI002_01342	hypothetical protein	Yes	Yes	No	Yes
1633	NSUI002_01543	hypothetical protein	Yes	Yes	No	Yes
1648	NSUI002_02257	phage protein	Yes	Yes	No	Yes

Cluster Number	Example Gene	Predicted Translated Product	Present also in			
			clade II	clade III	clade IV	clade V
1710	NSUI002_01959	helix-turn-helix, fis-type	Yes	Yes	No	Yes
1793	NSUI002_01553	hypothetical protein	Yes	Yes	No	Yes
1817	NSUI002_01550	phage protein	Yes	Yes	No	Yes
1859	NSUI002_01540	hypothetical protein	Yes	Yes	No	Yes
1860	NSUI002_01548	hypothetical protein	Yes	Yes	No	Yes
1887	NSUI002_01551	phage membrane protein	Yes	Yes	No	Yes
1896	NSUI002_01272	DNA-binding protein	Yes	Yes	No	Yes
1897	NSUI002_01552	hypothetical protein	Yes	Yes	No	Yes
1913	NSUI002_01542	YcfA-like protein	Yes	Yes	No	Yes
1914	NSUI002_01554	phage protein	Yes	Yes	No	Yes
1938	NSUI002_01947	hypothetical protein	Yes	Yes	No	Yes
1956	NSUI002_00970	glyoxalase/bleomycin resistance protein/dioxygenase superfamily protein	Yes	Yes	No	Yes
1973	NSUI002_02083	Phage infection protein	Yes	Yes	No	Yes
1974	NSUI002_02079	reticulocyte binding protein	Yes	Yes	No	Yes
1975	NSUI002_02065	membrane protein	Yes	Yes	No	Yes
1977	NSUI002_02071	reticulocyte binding protein	Yes	Yes	No	Yes
1981	NSUI002_02076	prophage function domain-containing protein	Yes	Yes	No	Yes
1982	NSUI002_02080	Ukp protein	Yes	Yes	No	Yes
1983	NSUI002_02078	hypothetical protein	Yes	Yes	No	Yes
1988	NSUI002_02064	hypothetical protein	Yes	Yes	No	Yes
1992	NSUI002_02067	hypothetical protein	Yes	Yes	No	Yes
1993	NSUI002_02063	hypothetical protein	Yes	Yes	No	Yes
1994	NSUI002_02061	hypothetical protein	Yes	Yes	No	Yes
1996	NSUI002_02074	hypothetical protein	Yes	Yes	No	Yes
1997	NSUI002_02075	putative lipoprotein	Yes	Yes	No	Yes
2000	NSUI002_02082	type VII secretion protein EssA	Yes	Yes	No	Yes
2003	NSUI002_02070	hypothetical protein	Yes	Yes	No	Yes
2005	NSUI002_02073	merozoite surface protein 1	Yes	Yes	No	Yes
2006	NSUI002_02069	hypothetical protein	Yes	Yes	No	Yes
2007	NSUI002_02072	glycosyltransferase	Yes	Yes	No	Yes
2009	NSUI002_02068	hypothetical protein	Yes	Yes	No	Yes
2011	NSUI002_02084	virulence factor EsxA	Yes	Yes	No	Yes
2012	NSUI002_02077	D-3-phosphoglycerate dehydrogenase	Yes	Yes	No	Yes
2013	NSUI002_02062	DNA translocase FtsK	Yes	Yes	No	Yes
2015	NSUI002_02081	YukD superfamily protein	Yes	Yes	No	Yes
2018	NSUI002_01173	membrane protein	Yes	Yes	No	Yes
2020	NSUI002_02060	hypothetical protein	Yes	Yes	No	Yes

Cluster Number	Example Gene	Predicted Translated Product	Present also in			
			clade II	clade III	clade IV	clade V
1756	NSUI002_01829	xanthine/uracil/vitamin C permease	Yes	Yes	No	No
1989	NSUI002_02058	hypothetical protein	Yes	Yes	No	No
266	NSUI002_02161	PTS system ascorbate-specific transporter subunit IIC	Yes	No	Yes	Yes
950	NSUI002_01212	replication initiator protein A	Yes	No	Yes	Yes
1118	NSUI002_01389	DNA alkylation repair enzyme	Yes	No	Yes	Yes
1355	NSUI002_01207	protease	Yes	No	Yes	Yes
1463	NSUI002_01206	hypothetical protein	Yes	No	Yes	Yes
1612	NSUI002_01209	arsenate reductase	Yes	No	Yes	Yes
1656	NSUI002_00560	hypothetical protein	Yes	No	Yes	Yes
1785	NSUI002_00543	plasmid addiction system, toxin protein	Yes	No	Yes	Yes
1831	NSUI002_01208	hypothetical protein	Yes	No	Yes	Yes
1857	NSUI002_00542	RelB protein	Yes	No	Yes	Yes
1936	NSUI002_01213	hypothetical protein	Yes	No	Yes	Yes
1944	NSUI002_00581	transposase	Yes	No	Yes	Yes
1976	NSUI002_01176	site-specific recombinase	Yes	No	Yes	No
125	NSUI002_01185	tetracycline resistance protein tetM	Yes	No	No	Yes
1597	NSUI002_01186	TnpV	Yes	No	No	Yes
2014	NSUI002_01174	hypothetical protein	Yes	No	No	Yes
1379	NSUI002_01166	Signal recognition particle GTPase	Yes	No	No	No
1526	NSUI002_01167	transcriptional regulator	Yes	No	No	No
1984	NSUI002_01181	hypothetical protein	Yes	No	No	No
1986	NSUI002_01180	plasmid recombination protein Mob family protein	Yes	No	No	No
1990	NSUI002_01175	SNF2 family protein	Yes	No	No	No
2002	NSUI002_01183	putative cytoplasmic protein	Yes	No	No	No
2016	NSUI002_01178	putative cytoplasmic protein	Yes	No	No	No
2017	NSUI002_01177	hypothetical protein	Yes	No	No	No
198	NSUI002_01263	phosphotransferase system cellobiose-specific component IIC	No	Yes	Yes	Yes
245	NSUI002_01262	6-phospho-beta-galactosidase	No	Yes	Yes	Yes
381	NSUI002_01258	O-acetylhomoserine sulfhydrylase	No	Yes	Yes	Yes
762	NSUI002_01261	galactose mutarotase-like protein	No	Yes	Yes	Yes

Cluster Number	Example Gene	Predicted Translated Product	Present also in			
			clade II	clade III	clade IV	clade V
893	NSUI002_01983	DegV family protein	No	Yes	Yes	Yes
1248	NSUI002_01260	hypothetical protein	No	Yes	Yes	Yes
1400	NSUI002_01793	ABC transporter ATPase	No	Yes	Yes	Yes
1446	NSUI002_00658	phosphotransferase system, mannose/fructose/N-acetylgalactosamine-specific component IIB	No	Yes	Yes	Yes
1650	NSUI002_00483	HNH endonuclease	No	Yes	Yes	Yes
1722	NSUI002_01264	phosphotransferase system cellobiose-specific component IIA	No	Yes	Yes	Yes
1870	NSUI002_01160	orf 10 protein	No	Yes	Yes	Yes
872	NSUI002_01265	transcriptional antiterminator	No	Yes	No	Yes
2004	NSUI002_02066	hypothetical protein	No	Yes	No	Yes
505	NSUI002_00130	integrase	No	Yes	No	No
530	NSUI002_00134	FtsK/SpoIIIE family protein	No	Yes	No	No
551	NSUI002_00132	replication initiation factor	No	Yes	No	No
1565	NSUI002_00133	hypothetical protein	No	Yes	No	No
1716	NSUI002_00136	hypothetical protein	No	Yes	No	No
1834	NSUI002_00135	membrane protein	No	Yes	No	No
1889	NSUI002_00131	hypothetical protein	No	Yes	No	No
1740	NSUI002_01000	50S ribosomal protein L21	No	No	Yes	Yes

[1] Orthologs between all 5 clades (N=1795) are not listed.
