# Supplementary material for: Complex Population Structure and Virulence Differences among Serotype 2 Streptococcus suis Strains Belonging to Sequence Type 28
Source: PLoS One. 2015 Sep 16;10(9):e0137760. doi: 10.1371/journal.pone.0137760 (PMC4574206; doi:10.1371/journal.pone.0137760)
Supplement: S5 Table — (PDF) [file pone.0137760.s008.pdf]

S5 Table. Common ortholog gene clusters among clade II ST28 *Streptococcus suis* strains.[1]

Cluster Number	Example Gene	Predicted Translated Product	Present also in			
			clade I	clade III	clade IV	clade V
55	NSUI005_00649	formate acetyltransferase	Yes	Yes	Yes	No
177	NSUI005_01110	GTPase subunit of restriction endonuclease	Yes	Yes	Yes	No
360	NSUI005_00648	phosphotransferase system cellobiose-specific component IIC	Yes	Yes	Yes	No
514	NSUI005_00730	hypothetical protein	Yes	Yes	Yes	No
525	NSUI005_01323	membrane protein	Yes	Yes	Yes	No
543	NSUI005_00652	glycerol dehydrogenase	Yes	Yes	Yes	No
580	NSUI005_01479	CRISPR-associated protein Cas7	Yes	Yes	Yes	No
667	NSUI005_00645	transcriptional regulator	Yes	Yes	Yes	No
689	NSUI005_00651	glycerol dehydrogenase	Yes	Yes	Yes	No
714	NSUI005_01481	CRISPR-associated protein Cas1	Yes	Yes	Yes	No
804	NSUI005_00549	putative ATPase (AAA+ superfamily)	Yes	Yes	Yes	No
911	NSUI005_00643	pyruvate-formate lyase-activating enzyme	Yes	Yes	Yes	No
1052	NSUI005_00644	sugar metabolism transcriptional regulator	Yes	Yes	Yes	No
1108	NSUI005_00350	cobalt ABC transporter ATPase	Yes	Yes	Yes	No
1155	NSUI005_00650	fructose-6-phosphate aldolase	Yes	Yes	Yes	No
1290	NSUI005_01291	16S RNA methylase RsmC	Yes	Yes	Yes	No
1300	NSUI005_01236	IgA-specific zinc metalloproteinase	Yes	Yes	Yes	No
1451	NSUI005_01654	histone acetyltransferase HPA2-like acetyltransferase	Yes	Yes	Yes	No
1476	NSUI005_01993	NTP pyrophosphohydrolase including oxidative damage repair enzymes	Yes	Yes	Yes	No
1493	NSUI005_00369	putative lipoprotein	Yes	Yes	Yes	No
1618	NSUI005_00258	membrane protein	Yes	Yes	Yes	No
1698	NSUI005_01480	CRISPR-associated protein Cas2	Yes	Yes	Yes	No
1729	NSUI005_00646	phosphotransferase system cellobiose-specific component IIA	Yes	Yes	Yes	No
1730	NSUI005_01408	hypothetical protein	Yes	Yes	Yes	No
1752	NSUI005_00647	phosphotransferase system	Yes	Yes	Yes	No

Cluster Number	Example Gene	Predicted Translated Product	Present also in			
			clade I	clade III	clade IV	clade V
		cellobiose-specific component IIB				
1780	NSUI005_00289	membrane protein	Yes	Yes	Yes	No
1824	NSUI005_00487	hypothetical protein	Yes	Yes	Yes	No
1928	NSUI005_00636	pyridine nucleotide-disulfide oxidoreductase	Yes	Yes	Yes	No
1947	NSUI005_00413	hypothetical protein	Yes	Yes	Yes	No
1968	NSUI005_00490	hypothetical protein	Yes	Yes	Yes	No
147	NSUI005_01938	LPXTG cell wall surface protein	Yes	Yes	No	Yes
216	NSUI005_01621	LPXTG-motif cell wall anchor domain-containing protein	Yes	Yes	No	Yes
335	NSUI005_00230	phosphotransferase system cellobiose-specific component IIC	Yes	Yes	No	Yes
599	NSUI005_01259	integrase family protein	Yes	Yes	No	Yes
647	NSUI005_01252	tagatose 1,6-diphosphate aldolase	Yes	Yes	No	Yes
750	NSUI005_01253	tagatose-6-phosphate kinase	Yes	Yes	No	Yes
836	NSUI005_01532	DNA replication protein, putative	Yes	Yes	No	Yes
865	NSUI005_01531	GTP-binding protein	Yes	Yes	No	Yes
951	NSUI005_01257	hypothetical protein	Yes	Yes	No	Yes
1015	NSUI005_01256	sugar metabolism transcriptional regulator	Yes	Yes	No	Yes
1305	NSUI005_01260	hypothetical protein	Yes	Yes	No	Yes
1312	NSUI005_01165	parvulin-like peptidyl-prolyl isomerase	Yes	Yes	No	Yes
1343	NSUI005_01540	transcriptional regulator	Yes	Yes	No	Yes
1345	NSUI005_01118	ATP-binding membrane protein	Yes	Yes	No	Yes
1358	NSUI005_01941	adenylate kinase	Yes	Yes	No	Yes
1366	NSUI005_01529	phage protein	Yes	Yes	No	Yes
1380	NSUI005_01254	galactose-6-phosphate isomerase subunit LacB	Yes	Yes	No	Yes
1401	NSUI005_02004	small molecule binding protein	Yes	Yes	No	Yes
1465	NSUI005_01526	phage-like protein	Yes	Yes	No	Yes
1542	NSUI005_01255	ribose 5-phosphate isomerase RpiB	Yes	Yes	No	Yes
1567	NSUI005_00260	hypothetical protein	Yes	Yes	No	Yes
1590	NSUI005_01326	hypothetical protein	Yes	Yes	No	Yes
1633	NSUI005_01528	hypothetical protein	Yes	Yes	No	Yes
1648	NSUI005_02030	phage protein	Yes	Yes	No	Yes

Cluster Number	Example Gene	Predicted Translated Product	Present also in			
			clade I	clade III	clade IV	clade V
1710	NSUI005_01870	helix-turn-helix, fis-type	Yes	Yes	No	Yes
1793	NSUI005_01538	hypothetical protein	Yes	Yes	No	Yes
1817	NSUI005_01535	phage protein	Yes	Yes	No	Yes
1859	NSUI005_01525	hypothetical protein	Yes	Yes	No	Yes
1860	NSUI005_01533	hypothetical protein	Yes	Yes	No	Yes
1887	NSUI005_01536	phage membrane protein	Yes	Yes	No	Yes
1896	NSUI005_01258	DNA-binding protein	Yes	Yes	No	Yes
1897	NSUI005_01537	hypothetical protein	Yes	Yes	No	Yes
1913	NSUI005_01527	YcfA-like protein	Yes	Yes	No	Yes
1914	NSUI005_01539	phage protein	Yes	Yes	No	Yes
1938	NSUI005_01858	hypothetical protein	Yes	Yes	No	Yes
1956	NSUI005_01009	glyoxalase/bleomycin resistance protein/dioxygenase superfamily protein	Yes	Yes	No	Yes
1973	NSUI005_01976	Phage infection protein	Yes	Yes	No	Yes
1974	NSUI005_01972	reticulocyte binding protein	Yes	Yes	No	Yes
1975	NSUI005_01958	membrane protein	Yes	Yes	No	Yes
1977	NSUI005_01964	reticulocyte binding protein	Yes	Yes	No	Yes
1981	NSUI005_01969	prophage function domain-containing protein	Yes	Yes	No	Yes
1982	NSUI005_01973	Ukp protein	Yes	Yes	No	Yes
1983	NSUI005_01971	hypothetical protein	Yes	Yes	No	Yes
1988	NSUI005_01957	hypothetical protein	Yes	Yes	No	Yes
1992	NSUI005_01960	hypothetical protein	Yes	Yes	No	Yes
1993	NSUI005_01956	hypothetical protein	Yes	Yes	No	Yes
1994	NSUI005_01954	hypothetical protein	Yes	Yes	No	Yes
1996	NSUI005_01967	hypothetical protein	Yes	Yes	No	Yes
1997	NSUI005_01968	putative lipoprotein	Yes	Yes	No	Yes
2000	NSUI005_01975	type VII secretion protein EssA	Yes	Yes	No	Yes
2003	NSUI005_01963	hypothetical protein	Yes	Yes	No	Yes
2005	NSUI005_01966	merozoite surface protein 1	Yes	Yes	No	Yes
2006	NSUI005_01962	hypothetical protein	Yes	Yes	No	Yes
2007	NSUI005_01965	glycosyltransferase	Yes	Yes	No	Yes
2009	NSUI005_01961	hypothetical protein	Yes	Yes	No	Yes
2011	NSUI005_01977	virulence factor EsxA	Yes	Yes	No	Yes
2012	NSUI005_01970	D-3-phosphoglycerate dehydrogenase	Yes	Yes	No	Yes
2013	NSUI005_01955	DNA translocase FtsK	Yes	Yes	No	Yes
2015	NSUI005_01974	YukD superfamily protein	Yes	Yes	No	Yes
2018	NSUI005_01166	membrane protein	Yes	Yes	No	Yes
2020	NSUI005_01953	hypothetical protein	Yes	Yes	No	Yes

Cluster Number	Example Gene	Predicted Translated Product	Present also in			
			clade I	clade III	clade IV	clade V
1756	NSUI005_00317	xanthine/uracil/vitamin C permease	Yes	Yes	No	No
1989	NSUI005_01951	hypothetical protein	Yes	Yes	No	No
266	NSUI005_02064	PTS system ascorbate-specific transporter subunit IIC	Yes	No	Yes	Yes
950	NSUI005_01200	replication initiator protein A	Yes	No	Yes	Yes
1118	NSUI005_01373	DNA alkylation repair enzyme	Yes	No	Yes	Yes
1355	NSUI005_01194	protease	Yes	No	Yes	Yes
1463	NSUI005_01193	hypothetical protein	Yes	No	Yes	Yes
1612	NSUI005_01196	arsenate reductase	Yes	No	Yes	Yes
1656	NSUI005_00581	hypothetical protein	Yes	No	Yes	Yes
1785	NSUI005_00564	plasmid addiction system, toxin protein	Yes	No	Yes	Yes
1831	NSUI005_01195	hypothetical protein	Yes	No	Yes	Yes
1857	NSUI005_00563	RelB protein	Yes	No	Yes	Yes
1936	NSUI005_01201	hypothetical protein	Yes	No	Yes	Yes
1944	NSUI005_00602	transposase	Yes	No	Yes	Yes
1976	NSUI005_01169	site-specific recombinase	Yes	No	Yes	No
125	NSUI005_01178	tetracycline resistance protein tetM	Yes	No	No	Yes
1597	NSUI005_01179	TnpV	Yes	No	No	Yes
2014	NSUI005_01167	hypothetical protein	Yes	No	No	Yes
1379	NSUI005_01159	Signal recognition particle GTPase	Yes	No	No	No
1526	NSUI005_01160	transcriptional regulator	Yes	No	No	No
1984	NSUI005_01174	hypothetical protein	Yes	No	No	No
1986	NSUI005_01173	plasmid recombination protein Mob family protein	Yes	No	No	No
1990	NSUI005_01168	SNF2 family protein	Yes	No	No	No
2002	NSUI005_01176	putative cytoplasmic protein	Yes	No	No	No
2016	NSUI005_01171	putative cytoplasmic protein	Yes	No	No	No
2017	NSUI005_01170	hypothetical protein	Yes	No	No	No
152	NSUI005_01192	Type IV secretory pathway, VirD4 component	No	Yes	Yes	Yes
243	NSUI005_01188	Orf26	No	Yes	Yes	Yes
421	NSUI005_00384	NADH:flavin oxidoreductase/NADH oxidase	No	Yes	Yes	Yes
636	NSUI005_00236	nitrate/sulfonate/bicarbonate ABC transporter periplasmic protein	No	Yes	Yes	Yes
661	NSUI005_00381	NADPH:quinone reductase-dependent oxidoreductase	No	Yes	Yes	Yes

Cluster Number	Example Gene	Predicted Translated Product	Present also in			
			clade I	clade III	clade IV	clade V
841	NSUI005_00382	alpha/beta superfamily hydrolase/acyltransferase	No	Yes	Yes	Yes
930	NSUI005_00383	dehydrogenase	No	Yes	Yes	Yes
940	NSUI005_00235	ABC transporter	No	Yes	Yes	Yes
988	NSUI005_00234	ABC transporter	No	Yes	Yes	Yes
1031	NSUI005_02019	prophage antirepressor	No	Yes	Yes	Yes
1093	NSUI005_01860	N-acetylglucosamine-1-phosphodiester alpha-N-acetylglucosaminidase-like exopolysaccharide biosynthesis protein	No	Yes	Yes	Yes
1219	NSUI005_02020	phage protein	No	Yes	Yes	Yes
1236	NSUI005_02155	phage protein	No	Yes	Yes	Yes
1386	NSUI005_00389	glycosyl transferase, clade 2 family protein	No	Yes	Yes	Yes
1443	NSUI005_00377	membrane protein	No	Yes	Yes	Yes
1550	NSUI005_00380	transcriptional regulator	No	Yes	Yes	Yes
1560	NSUI005_00269	membrane protein	No	Yes	Yes	Yes
1574	NSUI005_02027	hypothetical protein	No	Yes	Yes	Yes
1664	NSUI005_00583	hypothetical protein	No	Yes	Yes	Yes
1767	NSUI005_00268	hypothetical protein	No	Yes	Yes	Yes
1839	NSUI005_00266	hypothetical protein	No	Yes	Yes	Yes
1852	NSUI005_01544	hypothetical protein	No	Yes	Yes	Yes
1911	NSUI005_00542	IS66-Spn1, transposase	No	Yes	Yes	Yes
1942	NSUI005_02029	hypothetical protein	No	Yes	Yes	Yes
402	NSUI005_01138	phage integrase family site specific recombinase	No	Yes	Yes	No
1552	NSUI005_02153	phage encoded ArpU family transcriptional regulator	No	Yes	Yes	No
1815	NSUI005_00015	sphingosine kinase and enzymes related to diacylglycerol kinase	No	Yes	Yes	No
1838	NSUI005_01139	hypothetical protein	No	Yes	Yes	No
2026	NSUI005_01952	hypothetical protein	No	Yes	No	Yes
1615	NSUI005_00254	membrane protein	No	Yes	No	No
1788	NSUI005_00255	hypothetical protein	No	Yes	No	No
400	NSUI005_00551	3-phosphoshikimate 1-carboxyvinyltransferase	No	No	Yes	Yes
917	NSUI005_01190	Orf23	No	No	Yes	Yes
1624	NSUI005_01189	Orf25	No	No	Yes	Yes
1849	NSUI005_01191	membrane protein	No	No	Yes	Yes
1933	NSUI005_01534	hypothetical protein	No	No	No	Yes
1999	NSUI005_01175	RNA polymerase ECF-type	No	No	No	No

Cluster Number	Example Gene	Predicted Translated Product	Present also in			
			clade I	clade III	clade IV	clade V
		sigma factor				

[1] Orthologs between all 5 clades (N=1795) are not listed.
