# Supplementary material for: Complex Population Structure and Virulence Differences among Serotype 2 Streptococcus suis Strains Belonging to Sequence Type 28
Source: PLoS One. 2015 Sep 16;10(9):e0137760. doi: 10.1371/journal.pone.0137760 (PMC4574206; doi:10.1371/journal.pone.0137760)
Supplement: S6 Table — (PDF) [file pone.0137760.s009.pdf]

S6 Table. Common ortholog gene clusters among clade III ST28 *Streptococcus suis* strains.[1]

Cluster Number	Example Gene	Predicted Translated Product	Present also in			
			clade I	clade II	clade IV	clade V
55	NSUI021_00393	formate acetyltransferase	Yes	Yes	Yes	No
177	NSUI021_00973	GTPase subunit of restriction endonuclease	Yes	Yes	Yes	No
360	NSUI021_00392	phosphotransferase system cellobiose-specific component IIC	Yes	Yes	Yes	No
514	NSUI021_00692	hypothetical protein	Yes	Yes	Yes	No
525	NSUI021_01226	membrane protein	Yes	Yes	Yes	No
543	NSUI021_00396	glycerol dehydrogenase	Yes	Yes	Yes	No
580	NSUI021_01380	CRISPR-associated protein Cas7	Yes	Yes	Yes	No
667	NSUI021_00389	transcriptional regulator	Yes	Yes	Yes	No
689	NSUI021_00395	glycerol dehydrogenase	Yes	Yes	Yes	No
714	NSUI021_01382	CRISPR-associated protein Cas1	Yes	Yes	Yes	No
804	NSUI021_00291	putative ATPase (AAA+ superfamily)	Yes	Yes	Yes	No
911	NSUI021_00387	pyruvate-formate lyase-activating enzyme	Yes	Yes	Yes	No
1052	NSUI021_00388	sugar metabolism transcriptional regulator	Yes	Yes	Yes	No
1108	NSUI021_00424	cobalt ABC transporter ATPase	Yes	Yes	Yes	No
1155	NSUI021_00394	fructose-6-phosphate aldolase	Yes	Yes	Yes	No
1290	NSUI021_01194	16S RNA methylase RsmC	Yes	Yes	Yes	No
1300	NSUI021_01138	IgA-specific zinc metalloproteinase	Yes	Yes	Yes	No
1451	NSUI021_01586	histone acetyltransferase HPA2-like acetyltransferase	Yes	Yes	Yes	No
1476	NSUI021_02042	NTP pyrophosphohydrolase including oxidative damage repair enzymes	Yes	Yes	Yes	No
1493	NSUI021_00443	putative lipoprotein	Yes	Yes	Yes	No
1618	NSUI021_00258	membrane protein	Yes	Yes	Yes	No
1698	NSUI021_01381	CRISPR-associated protein Cas2	Yes	Yes	Yes	No
1729	NSUI021_00390	phosphotransferase system cellobiose-specific component IIA	Yes	Yes	Yes	No
1730	NSUI021_01310	hypothetical protein	Yes	Yes	Yes	No
1752	NSUI021_00391	phosphotransferase system	Yes	Yes	Yes	No

Cluster Number	Example Gene	Predicted Translated Product	Present also in			
			clade I	clade II	clade IV	clade V
		cellobiose-specific component IIB				
1780	NSUI021_01683	membrane protein	Yes	Yes	Yes	No
1824	NSUI021_00560	hypothetical protein	Yes	Yes	Yes	No
1928	NSUI021_00380	pyridine nucleotide-disulfide oxidoreductase	Yes	Yes	Yes	No
1947	NSUI021_00486	hypothetical protein	Yes	Yes	Yes	No
1968	NSUI021_00563	hypothetical protein	Yes	Yes	Yes	No
147	NSUI021_01905	LPXTG cell wall surface protein	Yes	Yes	No	Yes
216	NSUI021_01554	LPXTG-motif cell wall anchor domain-containing protein	Yes	Yes	No	Yes
335	NSUI021_00230	phosphotransferase system cellobiose-specific component IIC	Yes	Yes	No	Yes
599	NSUI021_01161	integrase family protein	Yes	Yes	No	Yes
647	NSUI021_01154	tagatose 1,6-diphosphate aldolase	Yes	Yes	No	Yes
750	NSUI021_01155	tagatose-6-phosphate kinase	Yes	Yes	No	Yes
836	NSUI021_01432	DNA replication protein, putative	Yes	Yes	No	Yes
865	NSUI021_01431	GTP-binding protein	Yes	Yes	No	Yes
951	NSUI021_01159	hypothetical protein	Yes	Yes	No	Yes
1015	NSUI021_01158	sugar metabolism transcriptional regulator	Yes	Yes	No	Yes
1305	NSUI021_01162	hypothetical protein	Yes	Yes	No	Yes
1312	NSUI021_01068	parvulin-like peptidyl-prolyl isomerase	Yes	Yes	No	Yes
1343	NSUI021_01439	transcriptional regulator	Yes	Yes	No	Yes
1345	NSUI021_00981	ATP-binding membrane protein	Yes	Yes	No	Yes
1358	NSUI021_02093	adenylate kinase	Yes	Yes	No	Yes
1366	NSUI021_01429	phage protein	Yes	Yes	No	Yes
1380	NSUI021_01156	galactose-6-phosphate isomerase subunit LacB	Yes	Yes	No	Yes
1401	NSUI021_02031	small molecule binding protein	Yes	Yes	No	Yes
1465	NSUI021_01426	phage-like protein	Yes	Yes	No	Yes
1542	NSUI021_01157	ribose 5-phosphate isomerase RpiB	Yes	Yes	No	Yes
1567	NSUI021_00260	hypothetical protein	Yes	Yes	No	Yes
1590	NSUI021_01229	hypothetical protein	Yes	Yes	No	Yes
1633	NSUI021_01428	hypothetical protein	Yes	Yes	No	Yes
1648	NSUI021_02101	phage protein	Yes	Yes	No	Yes

Cluster Number	Example Gene	Predicted Translated Product	Present also in			
			clade I	clade II	clade IV	clade V
1710	NSUI021_01838	helix-turn-helix, fis-type	Yes	Yes	No	Yes
1793	NSUI021_01437	hypothetical protein	Yes	Yes	No	Yes
1817	NSUI021_01434	phage protein	Yes	Yes	No	Yes
1859	NSUI021_01425	hypothetical protein	Yes	Yes	No	Yes
1860	NSUI021_01433	hypothetical protein	Yes	Yes	No	Yes
1887	NSUI021_01435	phage membrane protein	Yes	Yes	No	Yes
1896	NSUI021_01160	DNA-binding protein	Yes	Yes	No	Yes
1897	NSUI021_01436	hypothetical protein	Yes	Yes	No	Yes
1913	NSUI021_01427	YcfA-like protein	Yes	Yes	No	Yes
1914	NSUI021_01438	phage protein	Yes	Yes	No	Yes
1938	NSUI021_01826	hypothetical protein	Yes	Yes	No	Yes
1956	NSUI021_00872	glyoxalase/bleomycin resistance protein/dioxygenase superfamily protein	Yes	Yes	No	Yes
1973	NSUI021_02059	Phage infection protein	Yes	Yes	No	Yes
1974	NSUI021_02063	reticulocyte binding protein	Yes	Yes	No	Yes
1975	NSUI021_02077	membrane protein	Yes	Yes	No	Yes
1977	NSUI021_02071	reticulocyte binding protein	Yes	Yes	No	Yes
1981	NSUI021_02066	prophage function domain-containing protein	Yes	Yes	No	Yes
1982	NSUI021_02062	Ukp protein	Yes	Yes	No	Yes
1983	NSUI021_02064	hypothetical protein	Yes	Yes	No	Yes
1988	NSUI021_02078	hypothetical protein	Yes	Yes	No	Yes
1992	NSUI021_02075	hypothetical protein	Yes	Yes	No	Yes
1993	NSUI021_02079	hypothetical protein	Yes	Yes	No	Yes
1994	NSUI021_02081	hypothetical protein	Yes	Yes	No	Yes
1996	NSUI021_02068	hypothetical protein	Yes	Yes	No	Yes
1997	NSUI021_02067	putative lipoprotein	Yes	Yes	No	Yes
2000	NSUI021_02060	type VII secretion protein EssA	Yes	Yes	No	Yes
2003	NSUI021_02072	hypothetical protein	Yes	Yes	No	Yes
2005	NSUI021_02069	merozoite surface protein 1	Yes	Yes	No	Yes
2006	NSUI021_02073	hypothetical protein	Yes	Yes	No	Yes
2007	NSUI021_02070	glycosyltransferase	Yes	Yes	No	Yes
2009	NSUI021_02074	hypothetical protein	Yes	Yes	No	Yes
2011	NSUI021_02058	virulence factor EsxA	Yes	Yes	No	Yes
2012	NSUI021_02065	D-3-phosphoglycerate dehydrogenase	Yes	Yes	No	Yes
2013	NSUI021_02080	DNA translocase FtsK	Yes	Yes	No	Yes
2015	NSUI021_02061	YukD superfamily protein	Yes	Yes	No	Yes
2018	NSUI021_01069	membrane protein	Yes	Yes	No	Yes
2020	NSUI021_02082	hypothetical protein	Yes	Yes	No	Yes

Cluster Number	Example Gene	Predicted Translated Product	Present also in			
			clade I	clade II	clade IV	clade V
1756	NSUI021_01710	xanthine/uracil/vitamin C permease	Yes	Yes	No	No
1989	NSUI021_02084	hypothetical protein	Yes	Yes	No	No
198	NSUI021_01150	phosphotransferase system cellobiose-specific component IIC	Yes	No	Yes	Yes
245	NSUI021_01149	6-phospho-beta-galactosidase	Yes	No	Yes	Yes
381	NSUI021_01146	O-acetylhomoserine sulfhydrylase	Yes	No	Yes	Yes
762	NSUI021_01148	galactose mutarotase-like protein	Yes	No	Yes	Yes
893	NSUI021_01842	DegV family protein	Yes	No	Yes	Yes
1248	NSUI021_01147	hypothetical protein	Yes	No	Yes	Yes
1400	NSUI021_01675	ABC transporter ATPase	Yes	No	Yes	Yes
1446	NSUI021_00628	phosphotransferase system, mannose/fructose/N-acetylgalactosamine-specific component IIB	Yes	No	Yes	Yes
1650	NSUI021_00576	HNH endonuclease	Yes	No	Yes	Yes
1722	NSUI021_01152	phosphotransferase system cellobiose-specific component IIA	Yes	No	Yes	Yes
1870	NSUI021_01057	orf 10 protein	Yes	No	Yes	Yes
872	NSUI021_01153	transcriptional antiterminator	Yes	No	No	Yes
2004	NSUI021_02076	hypothetical protein	Yes	No	No	Yes
505	NSUI021_00138	integrase	Yes	No	No	No
530	NSUI021_00142	FtsK/SpoIIIE family protein	Yes	No	No	No
551	NSUI021_00140	replication initiation factor	Yes	No	No	No
1565	NSUI021_00141	hypothetical protein	Yes	No	No	No
1716	NSUI021_00144	hypothetical protein	Yes	No	No	No
1834	NSUI021_00143	membrane protein	Yes	No	No	No
1889	NSUI021_00139	hypothetical protein	Yes	No	No	No
152	NSUI021_01095	Type IV secretory pathway, VirD4 component	No	Yes	Yes	Yes
243	NSUI021_01033	conjugal transfer protein	No	Yes	Yes	Yes
421	NSUI021_00458	NADH:flavin oxidoreductase/NADH oxidase	No	Yes	Yes	Yes
636	NSUI021_00236	nitrate/sulfonate/bicarbonate ABC transporter periplasmic protein	No	Yes	Yes	Yes
661	NSUI021_00455	NADPH:quinone reductase-dependent oxidoreductase	No	Yes	Yes	Yes

Cluster Number	Example Gene	Predicted Translated Product	Present also in			
			clade I	clade II	clade IV	clade V
841	NSUI021_00456	alpha/beta superfamily hydrolase/acyltransferase	No	Yes	Yes	Yes
930	NSUI021_00457	dehydrogenase	No	Yes	Yes	Yes
940	NSUI021_00235	ABC transporter	No	Yes	Yes	Yes
988	NSUI021_00234	ABC transporter	No	Yes	Yes	Yes
1031	NSUI021_02106	prophage antirepressor	No	Yes	Yes	Yes
1093	NSUI021_01828	N-acetylglucosamine-1-phosphodiester alpha-N-acetylglucosaminidase-like exopolysaccharide biosynthesis protein	No	Yes	Yes	Yes
1219	NSUI021_02105	phage protein	No	Yes	Yes	Yes
1236	NSUI021_02097	phage protein	No	Yes	Yes	Yes
1386	NSUI021_00463	glycosyl transferase, clade 2 family protein	No	Yes	Yes	Yes
1443	NSUI021_00451	membrane protein	No	Yes	Yes	Yes
1550	NSUI021_00454	transcriptional regulator	No	Yes	Yes	Yes
1560	NSUI021_00269	membrane protein	No	Yes	Yes	Yes
1574	NSUI021_02104	hypothetical protein	No	Yes	Yes	Yes
1664	NSUI021_00325	hypothetical protein	No	Yes	Yes	Yes
1767	NSUI021_00268	hypothetical protein	No	Yes	Yes	Yes
1839	NSUI021_00266	hypothetical protein	No	Yes	Yes	Yes
1852	NSUI021_01443	hypothetical protein	No	Yes	Yes	Yes
1911	NSUI021_00611	IS66-Spn1, transposase	No	Yes	Yes	Yes
1942	NSUI021_02102	hypothetical protein	No	Yes	Yes	Yes
402	NSUI021_01001	integrase	No	Yes	Yes	No
1552	NSUI021_01906	phage encoded ArpU family transcriptional regulator	No	Yes	Yes	No
1815	NSUI021_00015	sphingosine kinase and enzymes related to diacylglycerol kinase	No	Yes	Yes	No
1838	NSUI021_01002	hypothetical protein	No	Yes	Yes	No
2026	NSUI021_02083	hypothetical protein	No	Yes	No	Yes
1615	NSUI021_00254	membrane protein	No	Yes	No	No
1788	NSUI021_00255	hypothetical protein	No	Yes	No	No
877	NSUI021_02099	replication protein	No	No	Yes	Yes
886	NSUI021_02109	KilA domain-containing protein	No	No	Yes	Yes
1089	NSUI021_02108	putative DNA-binding phage protein	No	No	Yes	Yes
1853	NSUI021_02107	putative DNA-binding phage protein	No	No	Yes	Yes
1888	NSUI021_02096	hypothetical protein	No	No	Yes	Yes

Cluster Number	Example Gene	Predicted Translated Product	Present also in			
			clade I	clade II	clade IV	clade V
1962	NSUI021_01854	hypothetical protein	No	No	Yes	No
1	NSUI021_01024	helicase	No	No	No	No
29	NSUI021_01004	blood clade A- and B-cleaving endo-beta-galactosidase	No	No	No	No
51	NSUI021_01040	IgA-specific zinc metalloproteinase	No	No	No	No
73	NSUI021_01032	membrane protein	No	No	No	No
116	NSUI021_01006	F5/8 type C domain containing protein	No	No	No	No
143	NSUI021_01012	L-fucose isomerase	No	No	No	No
279	NSUI021_01007	alpha-L-fucosidase	No	No	No	No
280	NSUI021_01020	ImpB/MucB/SamB family protein	No	No	No	No
283	NSUI021_01013	L-fuculose kinase fucK	No	No	No	No
343	NSUI021_01016	type IV secretory pathway, VirD2 components (relaxase)	No	No	No	No
376	NSUI021_01010	bacterial extracellular solute-binding protein	No	No	No	No
651	NSUI021_01041	hypothetical protein	No	No	No	No
664	NSUI021_01026	site-specific DNA methylase	No	No	No	No
731	NSUI021_01009	sugar ABC transporter permease	No	No	No	No
779	NSUI021_01005	glucokinase	No	No	No	No
826	NSUI021_01035	membrane protein	No	No	No	No
848	NSUI021_01008	sugar ABC transporter permease	No	No	No	No
1008	NSUI021_01014	regulator of fucose operon	No	No	No	No
1106	NSUI021_01018	repressor protein	No	No	No	No
1163	NSUI021_01029	hypothetical protein	No	No	No	No
1188	NSUI021_01011	L-fuculose phosphate aldolase	No	No	No	No
1238	NSUI021_01023	conjugative transposon protein	No	No	No	No
1281	NSUI021_01030	membrane protein	No	No	No	No
1399	NSUI021_01017	conjugative transposon membrane protein	No	No	No	No
1468	NSUI021_01039	hypothetical protein	No	No	No	No
1482	NSUI021_00145	hypothetical protein	No	No	No	No
1638	NSUI021_01034	conjugative transposon membrane protein	No	No	No	No
1668	NSUI021_01021	hypothetical protein	No	No	No	No
1676	NSUI021_01015	conjugative transposon mobilization protein	No	No	No	No

Cluster Number	Example Gene	Predicted Translated Product	Present also in			
			clade I	clade II	clade IV	clade V
1727	NSUI021_01037	single-strand binding protein	No	No	No	No
1755	NSUI021_01022	hypothetical protein	No	No	No	No
1776	NSUI021_01089	abortive infection protein AbiGII	No	No	No	No
1858	NSUI021_01028	hypothetical protein	No	No	No	No
1874	NSUI021_01036	conjugative transposon membrane protein	No	No	No	No
1891	NSUI021_01031	hypothetical protein	No	No	No	No
1941	NSUI021_01088	abortive infection system Abi protein	No	No	No	No
1957	NSUI021_01019	hypothetical protein	No	No	No	No
2146	NSUI021_01996	PTS system ascorbate-specific transporter subunit IIC	No	No	No	No
2147	NSUI021_01995	PTS system ascorbate-specific transporter subunit IIC	No	No	No	No

[1] Orthologs between all 5 clades (N=1795) are not listed.
