# Supplementary material for: Complex Population Structure and Virulence Differences among Serotype 2 Streptococcus suis Strains Belonging to Sequence Type 28
Source: PLoS One. 2015 Sep 16;10(9):e0137760. doi: 10.1371/journal.pone.0137760 (PMC4574206; doi:10.1371/journal.pone.0137760)
Supplement: S7 Table — (PDF) [file pone.0137760.s010.pdf]

S7 Table. Common ortholog gene clusters among clade IV ST28 *Streptococcus suis* strains.[1]

Cluster Number	Example Gene	Predicted Translated Product	Present also in			
			clade I	clade II	clade III	clade V
55	NSUI011_00675	formate acetyltransferase	Yes	Yes	Yes	No
177	NSUI011_01047	GTPase subunit of restriction endonuclease	Yes	Yes	Yes	No
360	NSUI011_00674	phosphotransferase system cellobiose-specific component IIC	Yes	Yes	Yes	No
514	NSUI011_00768	hypothetical protein	Yes	Yes	Yes	No
525	NSUI011_01480	membrane protein	Yes	Yes	Yes	No
543	NSUI011_00678	glycerol dehydrogenase	Yes	Yes	Yes	No
580	NSUI011_01702	CRISPR-associated protein Cas7	Yes	Yes	Yes	No
667	NSUI011_00671	transcriptional regulator	Yes	Yes	Yes	No
689	NSUI011_00677	glycerol dehydrogenase	Yes	Yes	Yes	No
714	NSUI011_01704	CRISPR-associated protein Cas1	Yes	Yes	Yes	No
804	NSUI011_00574	putative ATPase (AAA+ superfamily)	Yes	Yes	Yes	No
911	NSUI011_00669	pyruvate-formate lyase-activating enzyme	Yes	Yes	Yes	No
1052	NSUI011_00670	sugar metabolism transcriptional regulator	Yes	Yes	Yes	No
1108	NSUI011_00376	cobalt ABC transporter ATPase	Yes	Yes	Yes	No
1155	NSUI011_00676	fructose-6-phosphate aldolase	Yes	Yes	Yes	No
1290	NSUI011_01448	16S RNA methylase RsmC	Yes	Yes	Yes	No
1300	NSUI011_01201	IgA-specific zinc metalloproteinase	Yes	Yes	Yes	No
1451	NSUI011_01910	histone acetyltransferase HPA2-like acetyltransferase	Yes	Yes	Yes	No
1476	NSUI011_01303	NTP pyrophosphohydrolase including oxidative damage repair enzymes	Yes	Yes	Yes	No
1493	NSUI011_00395	putative lipoprotein	Yes	Yes	Yes	No
1618	NSUI011_00241	membrane protein	Yes	Yes	Yes	No
1698	NSUI011_01703	CRISPR-associated protein Cas2	Yes	Yes	Yes	No
1729	NSUI011_00672	phosphotransferase system cellobiose-specific component IIA	Yes	Yes	Yes	No
1730	NSUI011_01564	hypothetical protein	Yes	Yes	Yes	No
1752	NSUI011_00673	phosphotransferase system	Yes	Yes	Yes	No

Cluster Number	Example Gene	Predicted Translated Product	Present also in			
			clade I	clade II	clade III	clade V
		cellobiose-specific component IIB				
1780	NSUI011_01935	membrane protein	Yes	Yes	Yes	No
1824	NSUI011_00514	hypothetical protein	Yes	Yes	Yes	No
1928	NSUI011_00662	pyridine nucleotide-disulfide oxidoreductase	Yes	Yes	Yes	No
1947	NSUI011_00439	hypothetical protein	Yes	Yes	Yes	No
1968	NSUI011_00517	hypothetical protein	Yes	Yes	Yes	No
266	NSUI011_01350	PTS system ascorbate-specific transporter subunit IIC	Yes	Yes	No	Yes
950	NSUI011_01165	replication initiator protein A	Yes	Yes	No	Yes
1118	NSUI011_01530	DNA alkylation repair enzyme	Yes	Yes	No	Yes
1355	NSUI011_01160	protease	Yes	Yes	No	Yes
1463	NSUI011_01159	hypothetical protein	Yes	Yes	No	Yes
1612	NSUI011_01162	arsenate reductase	Yes	Yes	No	Yes
1656	NSUI011_00606	hypothetical protein	Yes	Yes	No	Yes
1785	NSUI011_00589	plasmid addiction system, toxin protein	Yes	Yes	No	Yes
1831	NSUI011_01161	hypothetical protein	Yes	Yes	No	Yes
1857	NSUI011_00588	RelB protein	Yes	Yes	No	Yes
1936	NSUI011_01166	hypothetical protein	Yes	Yes	No	Yes
1944	NSUI011_00627	transposase	Yes	Yes	No	Yes
1976	NSUI011_01133	site-specific recombinase	Yes	Yes	No	No
198	NSUI011_01214	phosphotransferase system cellobiose-specific component IIC	Yes	No	Yes	Yes
245	NSUI011_01213	6-phospho-beta-galactosidase	Yes	No	Yes	Yes
381	NSUI011_01210	O-acetylhomoserine sulfhydrylase	Yes	No	Yes	Yes
762	NSUI011_01212	galactose mutarotase-like protein	Yes	No	Yes	Yes
893	NSUI011_02092	DegV family protein	Yes	No	Yes	Yes
1248	NSUI011_01211	hypothetical protein	Yes	No	Yes	Yes
1400	NSUI011_01927	ABC transporter ATPase	Yes	No	Yes	Yes
1446	NSUI011_00703	phosphotransferase system, mannose/fructose/N-acetylgalactosamine-specific component IIB	Yes	No	Yes	Yes
1650	NSUI011_00531	HNH endonuclease	Yes	No	Yes	Yes
1722	NSUI011_01215	phosphotransferase system cellobiose-specific component IIA	Yes	No	Yes	Yes

Cluster Number	Example Gene	Predicted Translated Product	Present also in			
			clade I	clade II	clade III	clade V
1870	NSUI011_01121	ORF 10 protein	Yes	No	Yes	Yes
1740	NSUI011_00973	50S ribosomal protein L21	Yes	No	No	Yes
152	NSUI011_01158	Type IV secretory pathway, VirD4 component	No	Yes	Yes	Yes
243	NSUI011_01154	Orf26	No	Yes	Yes	Yes
421	NSUI011_00410	NADH:flavin oxidoreductase/NADH oxidase	No	Yes	Yes	Yes
636	NSUI011_00218	nitrate/sulfonate/bicarbonate ABC transporter periplasmic protein	No	Yes	Yes	Yes
661	NSUI011_00407	NADPH:quinone reductase-dependent oxidoreductase	No	Yes	Yes	Yes
841	NSUI011_00408	alpha/beta superfamily hydrolase/acyltransferase	No	Yes	Yes	Yes
930	NSUI011_00409	dehydrogenase	No	Yes	Yes	Yes
940	NSUI011_00217	ABC transporter	No	Yes	Yes	Yes
988	NSUI011_00216	ABC transporter	No	Yes	Yes	Yes
1031	NSUI011_02166	prophage antirepressor	No	Yes	Yes	Yes
1093	NSUI011_02078	N-acetylglucosamine-1-phosphodiester alpha-N-acetylglucosaminidase-like exopolysaccharide biosynthesis protein	No	Yes	Yes	Yes
1219	NSUI011_02165	phage protein	No	Yes	Yes	Yes
1236	NSUI011_02157	phage protein	No	Yes	Yes	Yes
1386	NSUI011_00415	glycosyl transferase, clade 2 family protein	No	Yes	Yes	Yes
1443	NSUI011_00403	membrane protein	No	Yes	Yes	Yes
1550	NSUI011_00406	transcriptional regulator	No	Yes	Yes	Yes
1560	NSUI011_00258	membrane protein	No	Yes	Yes	Yes
1574	NSUI011_02164	hypothetical protein	No	Yes	Yes	Yes
1664	NSUI011_00608	hypothetical protein	No	Yes	Yes	Yes
1767	NSUI011_00257	hypothetical protein	No	Yes	Yes	Yes
1839	NSUI011_00255	hypothetical protein	No	Yes	Yes	Yes
1852	NSUI011_01766	hypothetical protein	No	Yes	Yes	Yes
1911	NSUI011_00567	IS66-Spn1, transposase	No	Yes	Yes	Yes
1942	NSUI011_02162	hypothetical protein	No	Yes	Yes	Yes
402	NSUI011_01075	integrase	No	Yes	Yes	No
1552	NSUI011_01439	phage encoded ArpU family transcriptional regulator	No	Yes	Yes	No
1815	NSUI011_00015	sphingosine kinase and enzymes related to	No	Yes	Yes	No

Cluster Number	Example Gene	Predicted Translated Product	Present also in			
			clade I	clade II	clade III	clade V
		diacylglycerol kinase				
1838	NSUI011_01076	hypothetical protein	No	Yes	Yes	No
400	NSUI011_00576	3-phosphoshikimate 1-carboxyvinyltransferase	No	Yes	No	Yes
917	NSUI011_01156	Orf23	No	Yes	No	Yes
1624	NSUI011_01155	Orf25	No	Yes	No	Yes
1849	NSUI011_01157	membrane protein	No	Yes	No	Yes
877	NSUI011_02159	replication protein	No	No	Yes	Yes
886	NSUI011_02169	KilA domain-containing protein	No	No	Yes	Yes
1089	NSUI011_02168	putative DNA-binding phage protein	No	No	Yes	Yes
1853	NSUI011_02167	putative DNA-binding phage protein	No	No	Yes	Yes
1888	NSUI011_02156	hypothetical protein	No	No	Yes	Yes
1962	NSUI011_02104	hypothetical protein	No	No	Yes	No
1987	NSUI011_00277	IS630-Spn1, transposase Orf2	No	No	No	No

[1] Orthologs between all 5 clades (N=1795) are not listed.
