# Supplementary material for: Complex Population Structure and Virulence Differences among Serotype 2 Streptococcus suis Strains Belonging to Sequence Type 28
Source: PLoS One. 2015 Sep 16;10(9):e0137760. doi: 10.1371/journal.pone.0137760 (PMC4574206; doi:10.1371/journal.pone.0137760)
Supplement: S8 Table — (PDF) [file pone.0137760.s011.pdf]

S8 Table. Common ortholog gene clusters among clade V ST28 *Streptococcus suis* strains.[1]

Cluster Number	Example Gene	Predicted Translated Product	Present also in			
			clade I	clade II	clade III	clade IV
147	NSUI010_01827	LPXTG cell wall surface protein	Yes	Yes	Yes	No
216	NSUI010_01475	LPXTG-motif cell wall anchor domain-containing protein	Yes	Yes	Yes	No
335	NSUI010_00231	phosphotransferase system cellobiose-specific component IIC	Yes	Yes	Yes	No
599	NSUI010_01076	integrase family protein	Yes	Yes	Yes	No
647	NSUI010_01069	tagatose 1,6-diphosphate aldolase	Yes	Yes	Yes	No
750	NSUI010_01070	tagatose-6-phosphate kinase	Yes	Yes	Yes	No
836	NSUI010_01351	DNA replication protein, putative	Yes	Yes	Yes	No
865	NSUI010_01350	GTP-binding protein	Yes	Yes	Yes	No
951	NSUI010_01074	hypothetical protein	Yes	Yes	Yes	No
1015	NSUI010_01073	sugar metabolism transcriptional regulator	Yes	Yes	Yes	No
1305	NSUI010_01077	hypothetical protein	Yes	Yes	Yes	No
1312	NSUI010_01903	parvulin-like peptidyl-prolyl isomerase	Yes	Yes	Yes	No
1343	NSUI010_01359	transcriptional regulator	Yes	Yes	Yes	No
1345	NSUI010_01000	ATP-binding membrane protein	Yes	Yes	Yes	No
1358	NSUI010_01830	adenylate kinase	Yes	Yes	Yes	No
1366	NSUI010_01348	phage protein	Yes	Yes	Yes	No
1380	NSUI010_01071	galactose-6-phosphate isomerase subunit LacB	Yes	Yes	Yes	No
1401	NSUI010_01955	small molecule binding protein	Yes	Yes	Yes	No
1465	NSUI010_01345	phage-like protein	Yes	Yes	Yes	No
1542	NSUI010_01072	ribose 5-phosphate isomerase RpiB	Yes	Yes	Yes	No
1567	NSUI010_00271	hypothetical protein	Yes	Yes	Yes	No
1590	NSUI010_01143	hypothetical protein	Yes	Yes	Yes	No
1633	NSUI010_01347	hypothetical protein	Yes	Yes	Yes	No
1648	NSUI010_02088	phage protein	Yes	Yes	Yes	No
1710	NSUI010_01759	helix-turn-helix, fis-type	Yes	Yes	Yes	No
1793	NSUI010_01357	hypothetical protein	Yes	Yes	Yes	No
1817	NSUI010_01354	phage protein	Yes	Yes	Yes	No
1859	NSUI010_01344	hypothetical protein	Yes	Yes	Yes	No
1860	NSUI010_01352	hypothetical protein	Yes	Yes	Yes	No
1887	NSUI010_01355	phage membrane protein	Yes	Yes	Yes	No

Cluster Number	Example Gene	Predicted Translated Product	Present also in			
			clade I	clade II	clade III	clade IV
1896	NSUI010_01075	DNA-binding protein	Yes	Yes	Yes	No
1897	NSUI010_01356	hypothetical protein	Yes	Yes	Yes	No
1913	NSUI010_01346	YcfA-like protein	Yes	Yes	Yes	No
1914	NSUI010_01358	phage protein	Yes	Yes	Yes	No
1938	NSUI010_01747	hypothetical protein	Yes	Yes	Yes	No
1956	NSUI010_00891	glyoxalase/bleomycin resistance protein/dioxygenase superfamily protein	Yes	Yes	Yes	No
1973	NSUI010_01865	Phage infection protein	Yes	Yes	Yes	No
1974	NSUI010_01861	reticulocyte binding protein	Yes	Yes	Yes	No
1975	NSUI010_01846	membrane protein	Yes	Yes	Yes	No
1977	NSUI010_01852	reticulocyte binding protein	Yes	Yes	Yes	No
1981	NSUI010_01857	prophage function domain-containing protein	Yes	Yes	Yes	No
1982	NSUI010_01862	Ukp protein	Yes	Yes	Yes	No
1983	NSUI010_01859	hypothetical protein	Yes	Yes	Yes	No
1988	NSUI010_01845	hypothetical protein	Yes	Yes	Yes	No
1992	NSUI010_01848	hypothetical protein	Yes	Yes	Yes	No
1993	NSUI010_01844	hypothetical protein	Yes	Yes	Yes	No
1994	NSUI010_01842	hypothetical protein	Yes	Yes	Yes	No
1996	NSUI010_01855	hypothetical protein	Yes	Yes	Yes	No
1997	NSUI010_01856	putative lipoprotein	Yes	Yes	Yes	No
2000	NSUI010_01864	type VII secretion protein EssA	Yes	Yes	Yes	No
2003	NSUI010_01851	hypothetical protein	Yes	Yes	Yes	No
2005	NSUI010_01854	merozoite surface protein 1	Yes	Yes	Yes	No
2006	NSUI010_01850	hypothetical protein	Yes	Yes	Yes	No
2007	NSUI010_01853	glycosyltransferase	Yes	Yes	Yes	No
2009	NSUI010_01849	hypothetical protein	Yes	Yes	Yes	No
2011	NSUI010_01866	virulence factor EsxA	Yes	Yes	Yes	No
2012	NSUI010_01858	D-3-phosphoglycerate dehydrogenase	Yes	Yes	Yes	No
2013	NSUI010_01843	DNA translocase FtsK	Yes	Yes	Yes	No
2015	NSUI010_01863	YukD superfamily protein	Yes	Yes	Yes	No
2018	NSUI010_01904	membrane protein	Yes	Yes	Yes	No
2020	NSUI010_01841	hypothetical protein	Yes	Yes	Yes	No
266	NSUI010_01990	PTS system ascorbate-specific transporter subunit IIC	Yes	Yes	No	Yes
950	NSUI010_01942	replication initiator protein A	Yes	Yes	No	Yes
1118	NSUI010_01190	DNA alkylation repair enzyme	Yes	Yes	No	Yes
1355	NSUI010_01937	protease	Yes	Yes	No	Yes
1463	NSUI010_01936	hypothetical protein	Yes	Yes	No	Yes

Cluster Number	Example Gene	Predicted Translated Product	Present also in			
			clade I	clade II	clade III	clade IV
1612	NSUI010_01939	arsenate reductase	Yes	Yes	No	Yes
1656	NSUI010_00554	hypothetical protein	Yes	Yes	No	Yes
1785	NSUI010_00537	plasmid addiction system, toxin protein	Yes	Yes	No	Yes
1831	NSUI010_01938	hypothetical protein	Yes	Yes	No	Yes
1857	NSUI010_00536	RelB protein	Yes	Yes	No	Yes
1936	NSUI010_01943	hypothetical protein	Yes	Yes	No	Yes
1944	NSUI010_00575	transposase	Yes	Yes	No	Yes
125	NSUI010_01909	tetracycline resistance protein tetM	Yes	Yes	No	No
1597	NSUI010_01910	TnpV	Yes	Yes	No	No
2014	NSUI010_01905	hypothetical protein	Yes	Yes	No	No
198	NSUI010_01066	phosphotransferase system cellobiose-specific component IIC	Yes	No	Yes	Yes
245	NSUI010_01065	6-phospho-beta-galactosidase	Yes	No	Yes	Yes
381	NSUI010_01061	O-acetylhomoserine sulfhydrylase	Yes	No	Yes	Yes
762	NSUI010_01063	galactose mutarotase-like protein	Yes	No	Yes	Yes
893	NSUI010_01763	DegV family protein	Yes	No	Yes	Yes
1248	NSUI010_01062	hypothetical protein	Yes	No	Yes	Yes
1400	NSUI010_01596	ABC transporter ATPase	Yes	No	Yes	Yes
1446	NSUI010_00643	phosphotransferase system, mannose/fructose/N-acetylgalactosamine-specific component IIB	Yes	No	Yes	Yes
1650	NSUI010_00480	HNH endonuclease	Yes	No	Yes	Yes
1722	NSUI010_01067	phosphotransferase system cellobiose-specific component IIA	Yes	No	Yes	Yes
1870	NSUI010_01896	orf 10 protein	Yes	No	Yes	Yes
872	NSUI010_01068	transcriptional antiterminator	Yes	No	Yes	No
2004	NSUI010_01847	hypothetical protein	Yes	No	Yes	No
1740	NSUI010_00920	50S ribosomal protein L21	Yes	No	No	Yes
152	NSUI010_01935	Type IV secretory pathway, VirD4 component	No	Yes	Yes	Yes
243	NSUI010_01931	Orf26	No	Yes	Yes	Yes
421	NSUI010_00361	NADH:flavin oxidoreductase/NADH oxidase	No	Yes	Yes	Yes
636	NSUI010_00238	nitrate/sulfonate/bicarbonate ABC transporter periplasmic	No	Yes	Yes	Yes

Cluster Number	Example Gene	Predicted Translated Product	Present also in			
			clade I	clade II	clade III	clade IV
		protein				
661	NSUI010_00358	NADPH:quinone reductase-dependent oxidoreductase	No	Yes	Yes	Yes
841	NSUI010_00359	alpha/beta superfamily hydrolase/acyltransferase	No	Yes	Yes	Yes
930	NSUI010_00360	dehydrogenase	No	Yes	Yes	Yes
940	NSUI010_00237	ABC transporter	No	Yes	Yes	Yes
988	NSUI010_00236	ABC transporter	No	Yes	Yes	Yes
1031	NSUI010_02093	prophage antirepressor	No	Yes	Yes	Yes
1093	NSUI010_01749	N-acetylglucosamine-1-phosphodiester alpha-N-acetylglucosaminidase-like exopolysaccharide biosynthesis protein	No	Yes	Yes	Yes
1219	NSUI010_02092	phage protein	No	Yes	Yes	Yes
1236	NSUI010_02084	phage protein	No	Yes	Yes	Yes
1386	NSUI010_00366	glycosyl transferase, clade 2 family protein	No	Yes	Yes	Yes
1443	NSUI010_00354	membrane protein	No	Yes	Yes	Yes
1550	NSUI010_00357	transcriptional regulator	No	Yes	Yes	Yes
1560	NSUI010_00280	membrane protein	No	Yes	Yes	Yes
1574	NSUI010_02091	hypothetical protein	No	Yes	Yes	Yes
1664	NSUI010_00556	hypothetical protein	No	Yes	Yes	Yes
1767	NSUI010_00279	hypothetical protein	No	Yes	Yes	Yes
1839	NSUI010_00277	hypothetical protein	No	Yes	Yes	Yes
1852	NSUI010_01363	hypothetical protein	No	Yes	Yes	Yes
1911	NSUI010_00515	IS66-Spn1, transposase	No	Yes	Yes	Yes
1942	NSUI010_02089	hypothetical protein	No	Yes	Yes	Yes
2026	NSUI010_01840	hypothetical protein	No	Yes	Yes	No
400	NSUI010_00524	3-phosphoshikimate 1-carboxyvinyltransferase	No	Yes	No	Yes
917	NSUI010_01933	Orf23	No	Yes	No	Yes
1624	NSUI010_01932	Orf25	No	Yes	No	Yes
1849	NSUI010_01934	membrane protein	No	Yes	No	Yes
1933	NSUI010_01353	hypothetical protein	No	Yes	No	No
877	NSUI010_02086	replication protein	No	No	Yes	Yes
886	NSUI010_02096	KilA domain-containing protein	No	No	Yes	Yes
1089	NSUI010_02095	putative DNA-binding phage protein	No	No	Yes	Yes
1853	NSUI010_02094	putative DNA-binding phage protein	No	No	Yes	Yes
1888	NSUI010_02083	hypothetical protein	No	No	Yes	Yes

Cluster Number	Example Gene	Predicted Translated Product	Present also in			
			clade I	clade II	clade III	clade IV
1041	NSUI010_01913	erythromycin ribosome methylase	No	No	No	No
1705	NSUI010_00299	transposase	No	No	No	No
1909	NSUI010_00604	transposase, ISSsu4, authentic frameshift	No	No	No	No
1960	NSUI010_01912	hypothetical protein	No	No	No	No
2021	NSUI010_01263	hypothetical protein	No	No	No	No
2269	NSUI010_00326	cobalt ABC transporter ATPase	No	No	No	No
2270	NSUI010_00325	cobalt ABC transporter permease	No	No	No	No
2271	NSUI010_00324	membrane protein	No	No	No	No

[1] Orthologs between all 5 clades (N=1795) are not listed.
